# Multicomponent Salts of the Antidiabetic Agent Saxagliptin: Hydration-Assisted Assembly and Lipophilic Aggregation with Carboxylic Acids

**DOI:** 10.1021/acsomega.5c13537

**Published:** 2026-03-17

**Authors:** Alice E. Colatrella, Vanshika Bhaniramka, Peter F. Gilbert, Josephine Bicknell, Maria Pascual-Pedro, Gonzalo Campillo-Alvarado

**Affiliations:** † Department of Chemistry, 6686 Reed College, 3203 SE Woodstock Blvd, Portland, Oregon 97202-8199, United States; ‡ Department of Chemistry, Georgetown University, 37th and O Streets NW, Washington, District of Columbia 20057-1227, United States; § School of Public Health, Oregon Health & Science University-Portland State University School of Public Health, 1810 SW 5th Avenue, Suite 510, Portland, Oregon 97201, United States

## Abstract

We report the formation of five new salts of saxagliptin (**SAX**, trade name: Onglyza), an active pharmaceutical ingredient (API) approved by the FDA for the treatment of type II diabetes mellitus. Salt formation is achieved by exploiting the ability of **SAX** to accept a proton from five pharmacologically acceptable carboxylic acids, isonicotinic, adipic, succinic, salicylic, and 5-sulfosalicylic acids, resulting in charge-assisted hydrogen bonds between the protonated amine of **SAX** and the carboxylate or sulfonate anion. Hydration is prevalent in four of the five multicomponent solids, contributing to the stabilization of distinct supramolecular architectures. The crystal structures determined by single-crystal X-ray diffraction are further supported by thermal analysis, Fourier transform infrared spectroscopy, and molecular modeling. This work provides design elements for developing multicomponent salts of **SAX** and related lipophilic, adamantane-bearing APIs.

## Graphical abstract

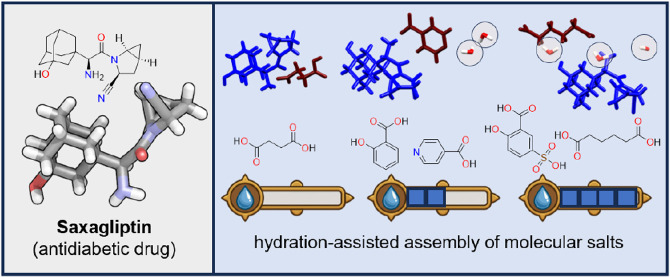

## 1 Introduction

Poor solubility of new chemical entities (NCEs) and pharmaceuticals presents major challenges for drug discovery and the biomedical industry.^1,2^ Approximately 40% of the drugs marketed in the United States are poorly soluble.^3^ The issue is particularly pronounced in highly lipophilic species (e.g., adamantane-bearing NCEs), which often require specific strategies to enhance solubility.^4,5^ The design of multicomponent systems, such as salts and cocrystals, has been shown to improve the solubility, bioavailability, and other physicochemical properties of poorly soluble active pharmaceutical ingredients (APIs) without altering their molecular structure.^6,7^ Pharmaceutical cocrystals are solids that incorporate two or more molecular components via weak, noncovalent interactions (e.g., hydrogen bonding, π–π stacking)^8–12^ while salts are formed through ionic interactions between two or more charged species.^13–15^ In both cases, the compounds used in combination with APIs (i.e., coformers) should ideally be nonpharmacologically injurious.

Saxagliptin (**SAX**, trade name: Onglyza) is an adamantane-bearing API with poor aqueous solubility and stability challenges. **SAX** is approved by the U.S. Food and Drug Administration (FDA) for the treatment of type II diabetes mellitus (T2DM), a condition characterized by the chronic inability of the body to produce sufficient insulin or respond appropriately to insulin, leading to elevated blood glucose levels.^16–18^ By acting as a competitive inhibitor of dipeptidyl peptidase IV (DPP-4), **SAX** prevents the enzymatic degradation of insulin-producing hormones^19–22^ resulting in increased blood glucose tolerance with rapid onset of action and sustained effects.^23–28^ However, the commercially available **SAX** free base monohydrate is prone to degradation, forming cyclic and epicyclic amidine impurities and a saxagliptin formyl amide impurity in solution^29,30^ and exhibits poor aqueous solubility.^31,32^ Notably, **SAX** has been reported to exhibit improved solid-state stability upon salt formation (e.g., hydrochloride, benzoate), as observed during formulation development and documented in patents.^29,33,34^

As part of our ongoing program on multicomponent solids, which focuses on the interaction landscape and properties of lipophilic APIs^35–39^ we describe here a series of new multicomponent salts based on **SAX** with pharmacologically acceptable carboxylic acids. The supramolecular architectures of the salts were determined by single-crystal X-ray diffraction (SCXRD). Specifically, **SAX** forms salts with isonicotinic (**INA**), adipic (**ADI**), succinic (**SUC**), 5-sulfosalicylic (**SSA**), and salicylic acids (**SAL**). The selected carboxylic acids were chosen based on their p*K*_*a*_ values, hydrogen-bonding propensity, and established use as pharmaceutically acceptable or listed by the FDA as Generally Recognized as Safe (GRAS). The present study aims to explore structural and supramolecular aspects of salt formation with **SAX** rather than to propose immediate therapeutic reformulations.

Notably, the resulting solids exhibit varying levels of hydration, affording 2­(**SAX**)·(**ADI**)·6­(H_2_O), 2­(**SAX**)·2­(**SAL**)·(H_2_O), 2­(**SAX**)·2­(**SSA**)·2­(H_2_O), and (**SAX**)·(**INA**)·2­(H_2_O), and (**SAX**)·(**SUC**). Our work is motivated by a patent by Gougoutas et al.^34^ describing **SAX** salts with hydrochloric, trifluoroacetic, benzoic, and fumaric acids. However, the structural landscape of multicomponent solids of **SAX** remains underexplored in the literature. Our structural observations are further corroborated by Fourier transform infrared spectroscopy (FT-IR), thermogravimetric analysis (TGA), ^1^H nuclear magnetic resonance (NMR) spectroscopy, and density functional theory (DFT) calculations. We anticipate that this work will inspire the design and synthesis of multicomponent solids of **SAX** and related hydrophilic APIs with improved pharmaceutical properties by providing structural and supramolecular design principles for salt and cocrystal engineering.Scheme 1

**Scheme 1:**
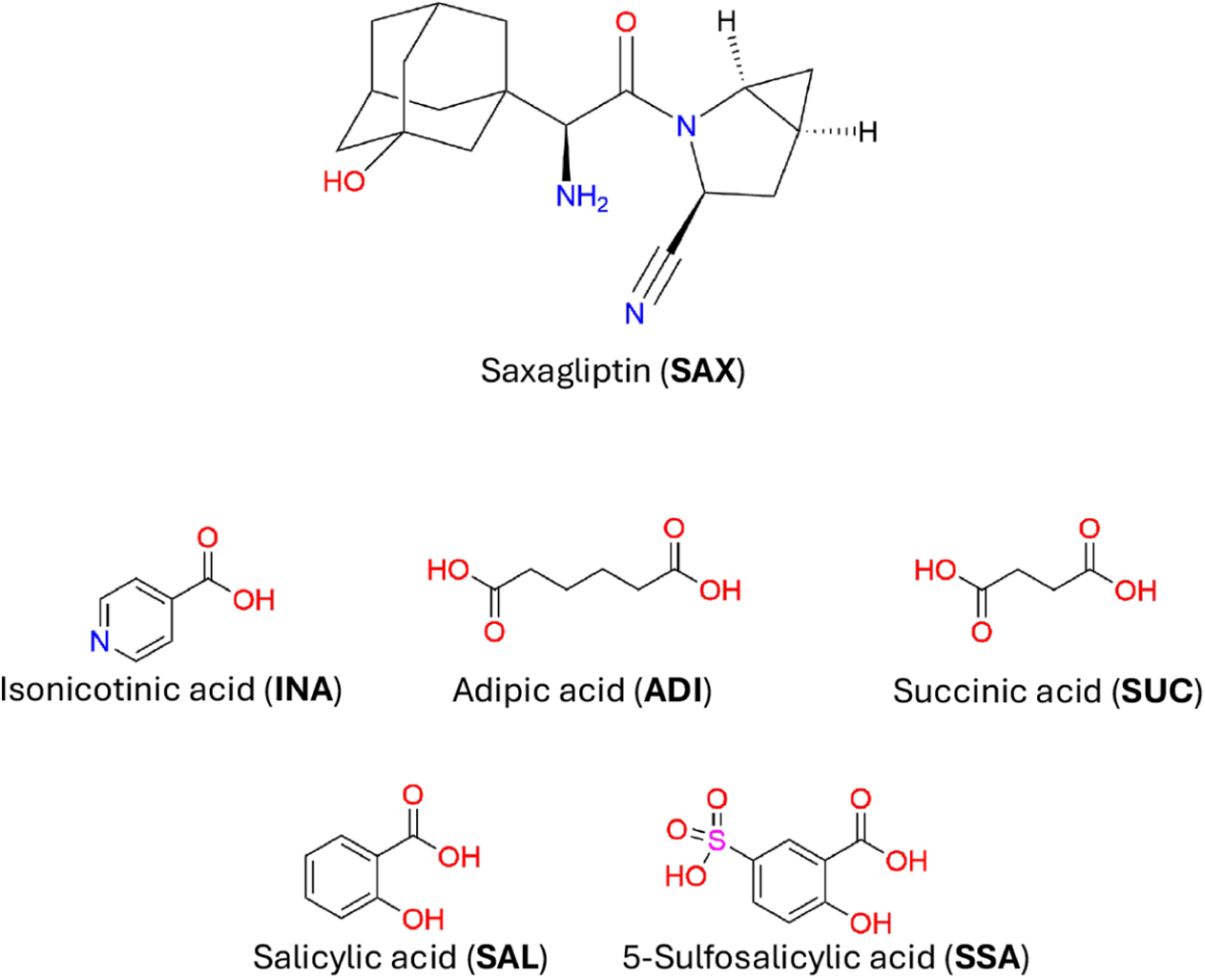
Molecular Diagram of the Saxagliptin (**SAX**) and the Salt Coformers: Isonicotinic, Adipic, Succinic, Salicylic, and 5-Sulfosalicylic Acids

## 2 Experimental Section

### 2.1 Crystal and Salt Syntheses

Methanol and acetonitrile were purchased from Sigma-Aldrich. Compounds **SUC**, **SAL**, **SSA**, **INA**, and **ADI** were purchased from Ambeed. **SAX** was purchased from Combi-Blocks. All chemicals were used as received without further purification. 2­(**SAX**)·(**ADI**)·6­(H_2_O), 2­(**SAX**)·2­(**SAL**)·(H_2_O), 2­(**SAX**)·2­(**SSA**)·2­(H_2_O), and (**SAX**)·(**INA**)·2­(H_2_O) were generated by the dissolution of **SAX** (0.125 mmol) with equimolar amounts of **SAL**, **SSA**, **INA**, or **ADI** (0.065 mmol) in 3 mL of methanol. Crystals of (**SAX**)·(**SUC**) were generated by heat and sonication-assisted dissolution of equimolar amounts of **SAX** (0.125 mmol) and **SUC** in acetonitrile (2 mL). Specific details for the formation of each system are included in the Supporting Information. Suitable single crystals for SCXRD were obtained by slow evaporation at room temperature for all samples, approximately 1 week after preparation.

All **SAX** salts were also synthesized by mechanochemical methods using a ball mill (Retsch Mixer Mill MM 400) by loading a 10 mL stainless steel jar with 0.321 mmol of **SAX**, the corresponding carboxylic acid coformer, and two drops of methanol, with the exception of (**SAX**)·(**SUC**), which required two drops of acetonitrile. The jar was equipped with 2 stainless-steel balls (7 mm), and milled at 30 Hz for 20 min. In all cases, the solid products were consistent with those obtained by solution crystallization, as confirmed by powder X-ray diffraction (PXRD) and ^1^H nuclear magnetic resonance (NMR) (see Supporting Information).

### 2.2 X-ray Crystallography and Instrumentation

SCXRD experiments for all samples were conducted using a Rigaku XtaLAB Mini II diffractometer with a CCD area detector (λ = 0.71073 Å, graphite monochromator). Collected data underwent standard data reduction and background correction from the integrated CrysAlisPro package. Structural refinement and solution were performed with Olex2,^40^ SHELXL,^41^ and SHELXT.^42^ Crystallographic data and selected metrics for the synthesized molecular salts are summarized in Tables S1–S7 (see Supporting Information). Water space calculations were conducted in Mercury using the CSD Materials Hydrate Analyzer tool using the default calculation settings.^43^ p*K*_*a*_ values were obtained using the p*K*_*a*_ calculator in the software MarvinSketch 18.13, p*K*_*a*_ Calculator Plugin by ChemAxon.^44–47^ PXRD data were collected on a Scintag XDS-2000 diffractometer using CuKα1 radiation (λ = 1.5418 Å). The samples were mounted and collected on glass slides, typically in the range of 5–40^°^ two-theta (scan type: step size: 0.02^°^, rate: 3 deg/min, continuous scan mode). The equipment was operated at 40 kV and 30 mA, and data were collected at room temperature. Fourier-transform infrared (FT-IR) spectra were recorded using a Thermo Fisher Scientific iS5 IR spectrometer from 600 to 4000 cm–1 using a diamond attenuated total reflectance (ATR) accessory. ^1^H NMR spectroscopy data were collected on a Bruker Ascend Evo 400 spectrometer and processed with MestReNova.^48^ Thermogravimetric analysis (TGA) data were collected on a Simultaneous Thermal Analyzer SDT650 under a nitrogen atmosphere with a 10 ^°^C/min ramp rate from 25^°^ to 300 ^°^C.

## 3 Results and Discussion

### 3.1 Rationale toward Design

To evaluate the feasibility of carboxylic acids **SUC**, **ADI**, **SAL**, **SSA** and **INA** as potential salt formers or coformers for **SAX** and predict intermolecular interactions, molecular electrostatic potential (ESP) maps were generated from DFT-optimized geometries (B3LYP/6-31G­(d,p)) via single-point calculations.^49–51^ Figure 1 depicts nucleophilic (i.e., electron-rich) regions in red and electrophilic (i.e., electron-deficient) regions in blue. Analysis of the ESP maps revealed that **SAX** has four potential hydrogen bond acceptor sites, with the primary amine showing the global minimum (−37 kcal/mol). The ESP maps of the carboxylic acids confirmed their potential as hydrogen bond donors, with **INA** and **SAL** presenting one carboxylic acid interaction site, and **SSA**, **SUC**, **ADI** presenting two interaction sites each, suitable for salt or cocrystal formation. The global maximum ESP sites in carboxylic acid motifs ranged from 46 to 53 kcal/mol, consistent with values reported for carboxylic acids in salts and cocrystals.^50^ Based on the analysis, we hypothesize the formation of salts or cocrystals with varying stoichiometries, as well as the potential inclusion of water or solvent molecules.^52^

**Figure 1:**
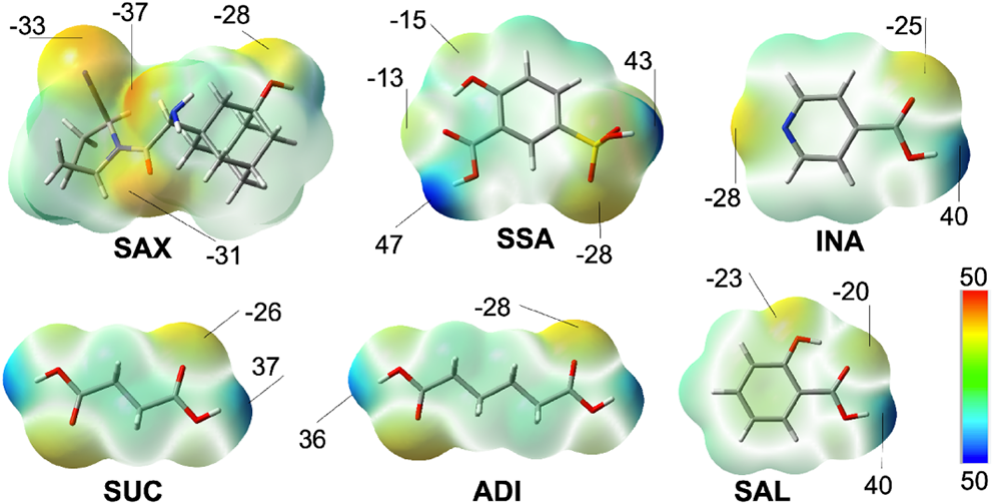
Electrostatic potential (ESP) maps of **SAX**, **SUC**, **ADI**, **SAL**, **SSA** and **INA**. Maxima and minima values in marked regions are given in kcal/mol.

When acid–base pairs are combined, there is a possibility for proton transfer to occur. Generally, it can be predicted that salt formation will occur if Δp*K*_*a*_ (Δp*K*_*a*_ = p*K*_*a*_(base^+^–H) – p*K*_*a*_(acid)) is greater than 4 using the p*K*_*a*_ rule.^53,54^ Conversely, when Δp*K*_*a*_ is less than 1, nonionic interactions, such as those in cocrystals, are typically expected. In this study, we selected five carboxylic acid molecules with p*K*_*a*_ values ranging from −2.81 (**SSA**) to 4.62 (**ADI**) to generate new multicomponent formulations with **SAX**. In all cases, we predict that molecular salts will be present in all resulting crystalline complexes (Table 1).^55^

**Table 1:** Calculated p*K_a_* Values for **SAX** and Carboxylic Acids (H-Donors), Δp*K_a_* Values, Predicted and Observed Outcomes

Carboxylic Acid	Donor p*K*_*a*_	**SAX** p*K*_*a*_	Δp*K*_*a*_	Predicted Outcome	Observed Outcome
**SUC**	3.55	7.90	4.35	Salt	Salt
**ADI**	4.62	7.90	3.28	Salt	Salt
**SAL**	2.79	7.90	5.11	Salt	Salt
**SSA**	–2.81	7.90	10.71	Salt	Salt
**INA**	2.01	7.90	5.89	Salt	Salt

### 3.2 X-ray Structures of Saxagliptin Salts

To test our hypothesis, **SAX** was dissolved with carboxylic acids **SUC**, **SSA**, **INA**, **ADI** and **SAL**, affording single crystals as colorless prisms. The formulations of (**SAX**)·(**SUC**), 2­(**SAX**)·(**ADI**)·6­(H_2_O), 2­(**SAX**)·2­(**SAL**)·(H_2_O), 2­(**SAX**)·2­(**SSA**)·2­(H_2_O), and (**SAX**)·(**INA**)·2­(H_2_O) were confirmed by SCXRD, PXRD, ^1^H NMR and FT-IR spectroscopies. SCXRD experiments demonstrated that the multicomponent solids crystallized as salts as predicted by the Δp*K*_*a*_ calculations.^45,55^ An overlay of the crystal structures revealed minor variations in the twist angles of the adamantanol cages and the methanoprolineamide motif relative to the central α-methine carbon (Figure S1, Supporting Information). The molecular conformations of **SAX** were found to be conserved across the series (root-mean-square deviations, RMSD < 0.41 Å), with the exception of the **SAL** salt (RMSD = 1.58 Å) (see Table S6 for details). This deviation is attributed to the rotation of the flexible adamantanol ring to enable hydrogen bonding with water.

In all cases, the presence of diagnostic broad bands in the 3300–3550 cm^–1^ region of FT-IR spectra was employed to confirm the stretching vibrations of the protonated amine in **SAX** (i.e., H–^+^NH_2_), consistent with charge-assisted hydrogen bonding in primary amine–carboxylate salts (see Table S7 for selected FT-IR absorption bands).^56,57^

#### 3.2.1 Analysis of (**SAX**)·(**SUC**) Salt

SCXRD analysis revealed the components in (**SAX**)·(**SUC**) to crystallize in the monoclinic space group *P*2_1_ with an asymmetric unit comprising one **SAX** unit and one **SUC** unit. The two-component assembly is supported by a [COO^–^···H–^+^NH_2_] charge-assisted hydrogen bond (Figure 2a). Notably, the **SUC** units are monodeprotonated and further stabilized through [COO^–^···H–OOC] hydrogen bonds between neighboring **SUC** units, forming zigzag chains along the *a*-axis (Figure S6). The interaction is akin to those reported in other polycarboxylic acid systems.^58,59^ Partial deprotonation of **SUC** (i.e., hemisuccinate) has also been used to generate solids of ciprofloxacin with multiple stoichiometries.^60^ In (**SAX**)·(**SUC**), the supramolecular architecture is further stabilized by [O_phenol_–H···O=C] hydrogen bonds between **SAX** and the neutral carboxylic acid site of **SUC**, as well as [C_adamantane_–H···O_phenol_] interactions between neighboring **SAX** units. Additional lipophilic aggregation arises from dispersion forces in van der Waals [H···H] contacts involving adamantane cages and methanoprolineamide motifs. Notably, the hydrogen bonding network is confined primarily to a hydrophilic region (Figure 2b), reminiscent of salt conglomerates of achiral carboxylic acids with chiral amines.^61^ Salt formation was further confirmed by FT-IR spectroscopy, which showed broad bands at 3273 and 3394 cm^–1^ (H–^+^NH_2_ stretch), characteristic of the formation of primary amine-carboxylic acid salts.^56,57^

**Figure 2:**
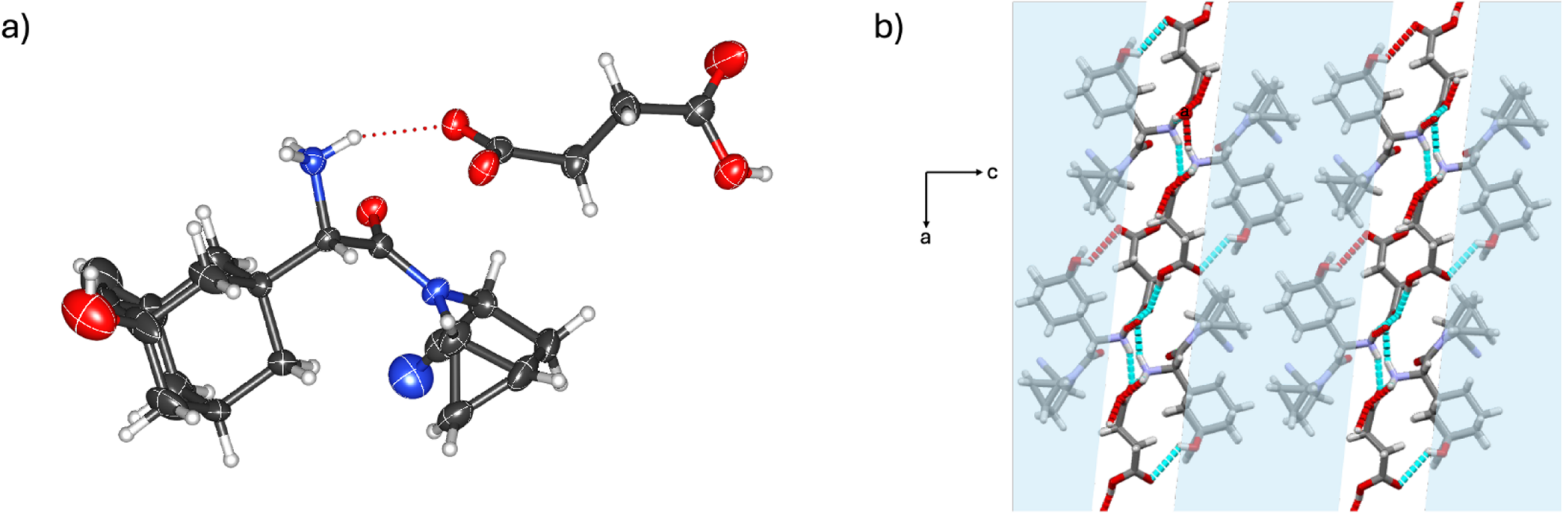
X-ray crystal structure of (**SAX**)·(**SUC**): (a) molecular units sustained by [COO^–^···H–^+^NH_2_] hydrogen bonds, and (b) lipophilic assemblies of **SAX** aggregates in the *ac*-plane.

#### 3.2.2 Analysis of 2­(**SAX**)·(**ADI**)·6­(H_2_O) Hydrate Salt

The components of 2­(**SAX**)·(**ADI**)·6­(H_2_O) crystallize in the monoclinic space group *I*2 with an asymmetric unit that contains one **SAX** unit, one-half of an **ADI** unit, and three H_2_O molecules. In contrast to (**SAX**)·(**SUC**), which features a shorter dicarboxylic acid, **ADI** in 2­(**SAX**)·(**ADI**)·6­(H_2_O) is fully ionized, resulting in a three-component assembly sustained by two [COO^–^···H–^+^NH_2_] hydrogen bonds between **ADI** and the amine group of two **SAX** units (Figure 3a and Figure S2), in addition to six H_2_O molecules. A 2-fold rotoinversion axis is positioned along the **ADI** unit as previously observed in fully deprotonated diacids.^62^ The adipate ion has also been shown to support the formation of a salbutamol salt cocrystal via protonation of a secondary amine group.^62^ In 2­(**SAX**)·(**ADI**)·6­(H_2_O), adamantyl cages in adjacent assemblies van der Waals [H···H] contacts, forming lipophilic aggregations. Notably, water molecules stabilize the **SAX** and **ADI** units through square-shaped channels sustained with alternating [H_2_O···H–^+^NH_2_] and [H_2_O···H–O_phenol_] hydrogen bonds (Figure 3b,c and Figure S2). Additional structural support is provided by [COO^–^···H–OH] interactions. Hydrate analysis shows that water molecules occupy 11.8% of the total unit cell volume, which is the highest hydration level observed in the series (Figure 3d). The hydrogen bonding network is confined primarily to a hydrophilic region, similar to that observed in (**SAX**)·(**SUC**).

**Figure 3:**
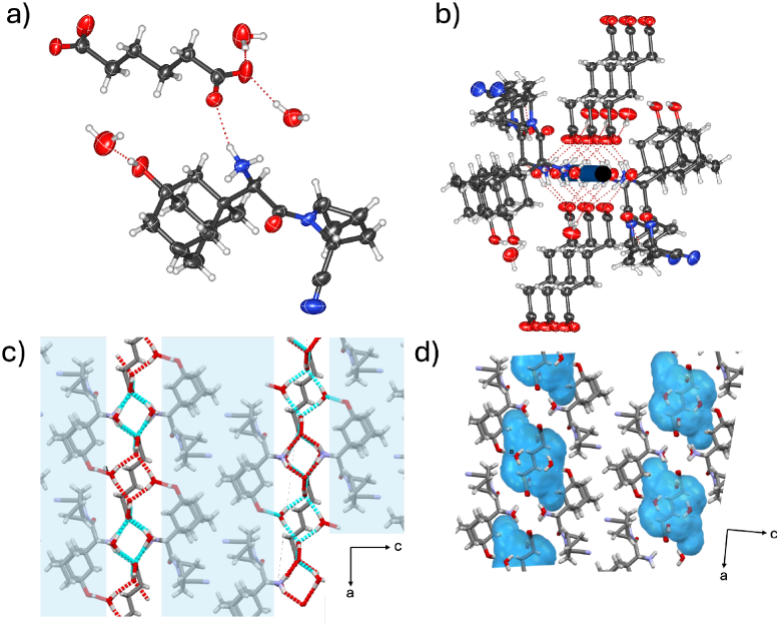
X-ray crystal structure of 2­(**SAX**)·(**ADI**)·6­(H_2_O): (a) molecular units connected by [COO^–^···H–^+^NH_2_] charge-assisted hydrogen bonds, (b) water molecules forming square-shaped channels sustained by [H_2_O···H–^+^NH_2_] and [COO^–^···H–OH] hydrogen bonds, (c) lipophilic regions of **SAX** aggregates in the *ac*-plane, and (d) calculated water space in the *ac*-plane.

#### 3.2.3 Analysis of 2­(**SAX**)·2­(**SAL**)·(H_2_O) Hydrate Salt

SCXRD analysis revealed the components in 2­(**SAX**)·2­(**SAL**)·(H_2_O) to crystallize in the monoclinic space group *P*2_1_ with an asymmetric unit that contains two **SAX** units, two **SAL** units, and one H_2_O molecule (Figure 4a). The **SAL** cations show intramolecular [COO^–^···H–O­(C)] hydrogen bonds, consistent with previously reported salts with **SAL**.^63–66^ In the 2­(**SAX**)·2­(**SAL**)·(H_2_O) solid, the primary four-component assembly is sustained by two [COO^–^···H–^+^NH_2_] charge-assisted hydrogen bonds between **SAX** and **SAL**, as well as [O_phenol_–H···O_phenol_] between adamantanol units bridging two **SAX** units. An additional [O_phenol_–H···OH_2_] bond involving the remaining adamantanol motif supports water inclusion and extends the hydrogen bonding network along the *a*-axis (Figure 4b and Figure S4). The sinusoidal architecture is supported by [C–H···π] contacts^67,68^ between **SAX** and **SAL** components (Figure 4c). Hydrate analysis revealed that water molecules occupy 7.8% of the total unit cell volume (Figure 4d).

**Figure 4:**
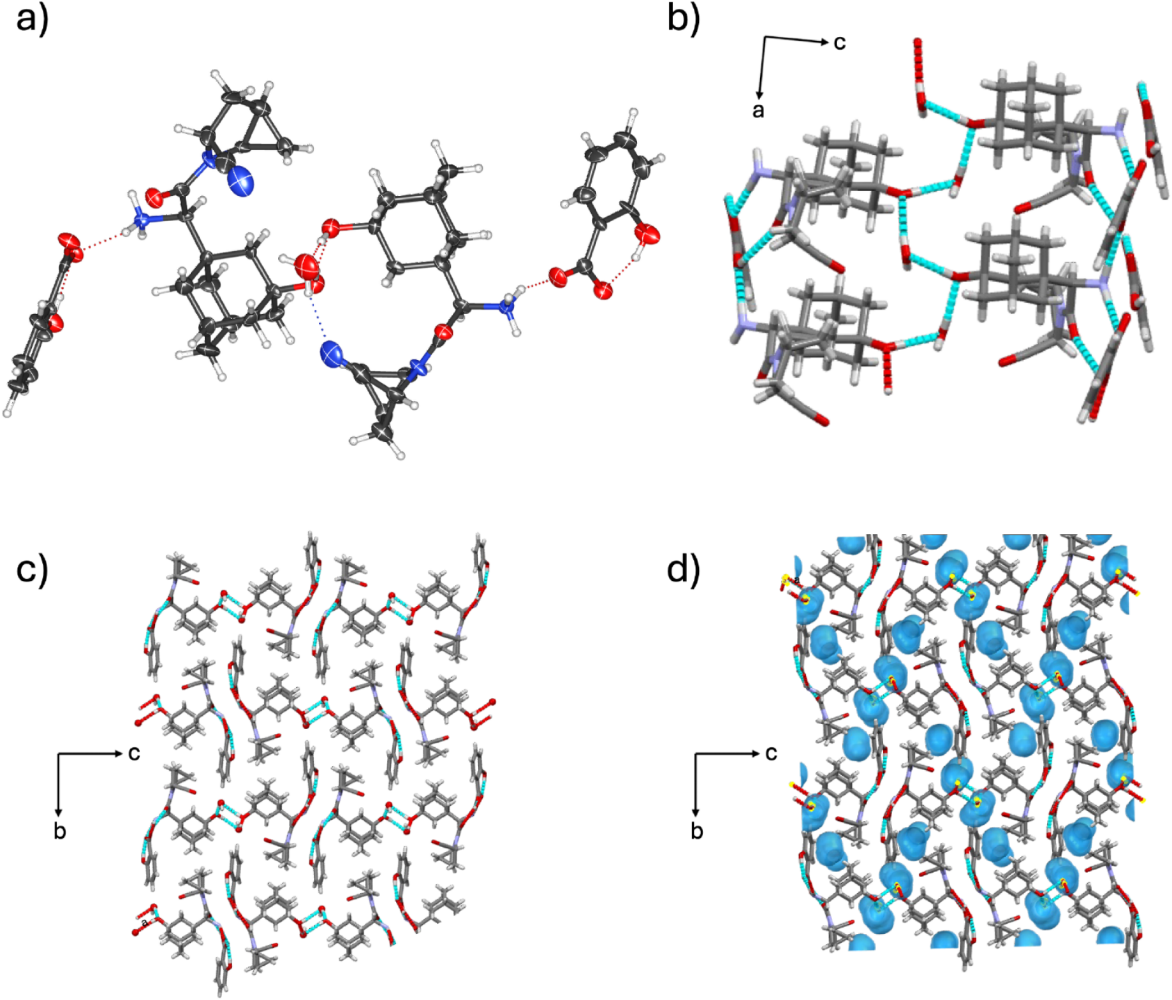
X-ray crystal structure of 2­(**SAX**)·2­(**SAL**)·(H_2_O): (a) molecular units connected by [COO^–^···H–^+^NH_2_] charge-assisted hydrogen bonds, (b) **SAX**–H_2_O hydrogen bonding network in the *ac*-plane, (c) extended view in the *bc*-plane showing a sinusoidal architecture, and (d) calculated water space in the *bc*-plane.

#### 3.2.4 Analysis of 2­(**SAX**)·2­(**SSA**)·2­(H_2_O) Hydrate Salt

The components in 2­(**SAX**)·2­(**SSA**)·2­(H_2_O) crystallize in the monoclinic space group *P*2_1_ with an asymmetric unit containing two **SAX**, two **SSA** units, and two water molecules. One **SAX** molecule is disordered over two positions in the adamantanol cage (Figure 5a). In contrast to the other **SAX** salts in the series, the six-component assembly is primarily sustained by [S–O^–^···H–^+^NH_2_] hydrogen bonds involving the sulfonate group, rather than a carboxylate. The solid-state behavior of **SSA** is consistent with reported salts of dapsone and tetrahydroberberine,^69,70^ in which deprotonation occurs only in the sulfonic acid group. In 2­(**SAX**)·2­(**SSA**)·2­(H_2_O), the protonated amine in **SAX** and the carbonyl group in **SSA** are further stabilized through [H_2_N^+^–H···OH_2_] hydrogen bonds with water, which also plays a role in sustaining the hydrogen-bonding network within the hydrophilic region (Figure S5). As in 2­(**SAX**)·(**ADI**)·6­(H_2_O), the hydrogen-bonding network is confined primarily to a defined hydrophilic domain. Hydrate analysis shows that water molecules occupy 1.4% of the total unit cell volume (Figure 5b,c). Meanwhile, adamantyl cages in adjacent assemblies exhibit van der Waals [H···H] contacts, forming lipophilic aggregations. Additional [O_phenol_–H···N_nitrile_] hydrogen bonds occur between the adamantanol cage and the methanoprolineamide motif of neighboring **SAX** units.

**Figure 5:**
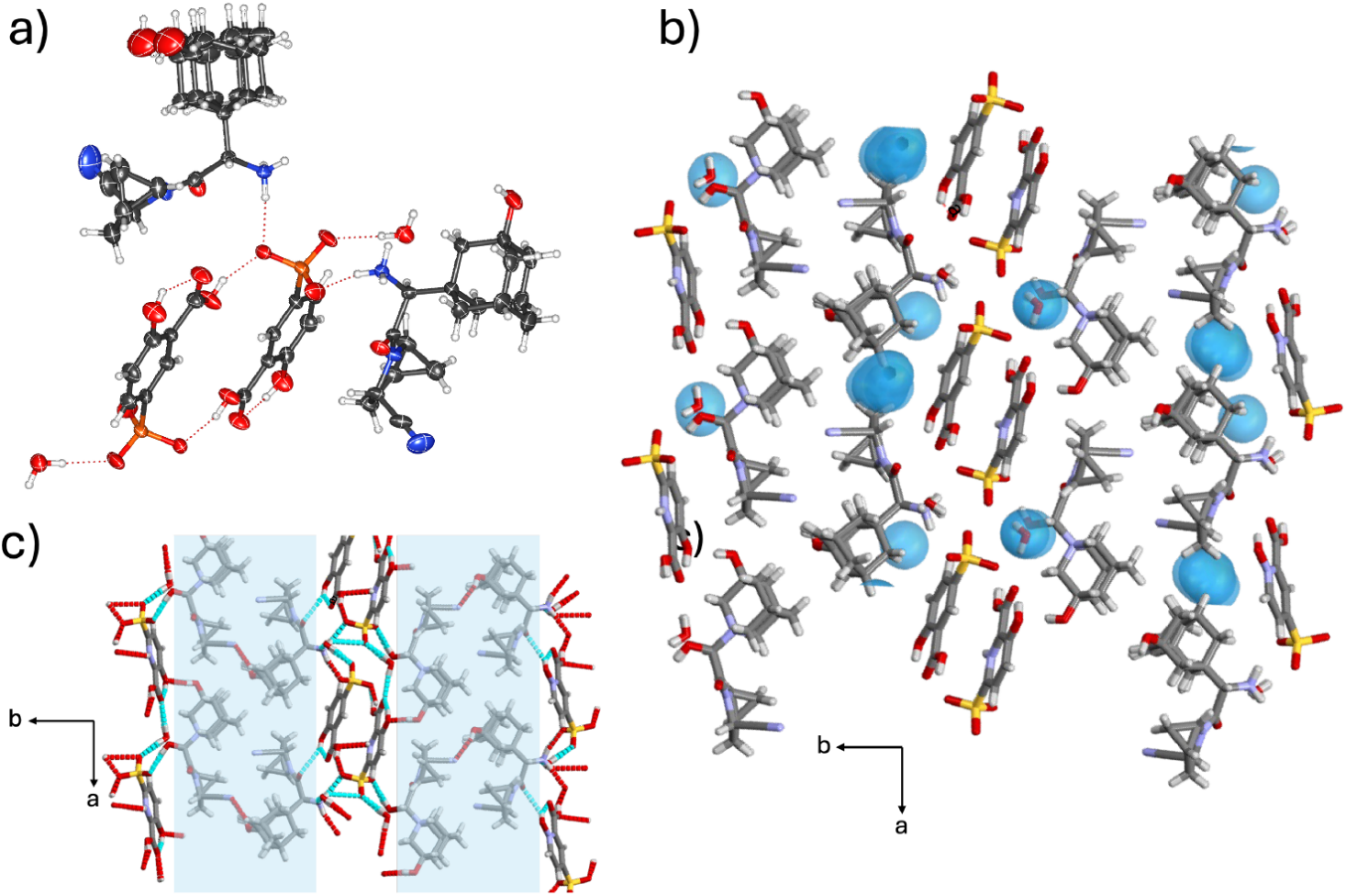
X-ray crystal structure of 2­(**SAX**)·2­(**SSA**)·2­(H_2_O): (a) molecular units connected by [S–O^–^···H–^+^NH_2_] charge-assisted hydrogen bonds, (b) lipophilic assemblies of **SAX** aggregates in the *ab*-plane, and (c) calculated water space in the *ab*-plane.

#### 3.2.5 Analysis of (**SAX**)·(**INA**)·2­(H_2_O) Hydrate Salt

The components of (**SAX**)·(**INA**)·2­(H_2_O) crystallize in the monoclinic space group *P*2_1_ with an asymmetric unit containing one **SAX**-**INA** pair and two water molecules. The four-component assembly is primarily supported by charge-assisted [COO^–^···H–^+^NH_2_] hydrogen bonds (Figure 6a) between the deprotonated acid in **INA** and the protonated amine in **SAX**, consistent with the other **SAX** salts in the series. Adamantyl cages in adjacent assemblies exhibit van der Waals [H···H] contacts, forming lipophilic aggregations, which are further stabilized through weak [C_methanoprolineamide_–H···O=C] and [C_methanoprolineamide_–H····O_phenol_] interactions. The two water molecules are supported by [HO–H···N_pyridine_], [COO^–^···H–OH] and [H_2_O···H–^+^NH_2_] hydrogen bonds with the pyridine ring, carboxylate, and ammonium ion, respectively (Figure 6b,c and Figure S3). The interactions encapsulate the water molecules within discrete hydrogen-bonded pockets (Figure 6d). Hydrate analysis revealed that water molecules occupy 6.6% of the total cell volume.

**Figure 6:**
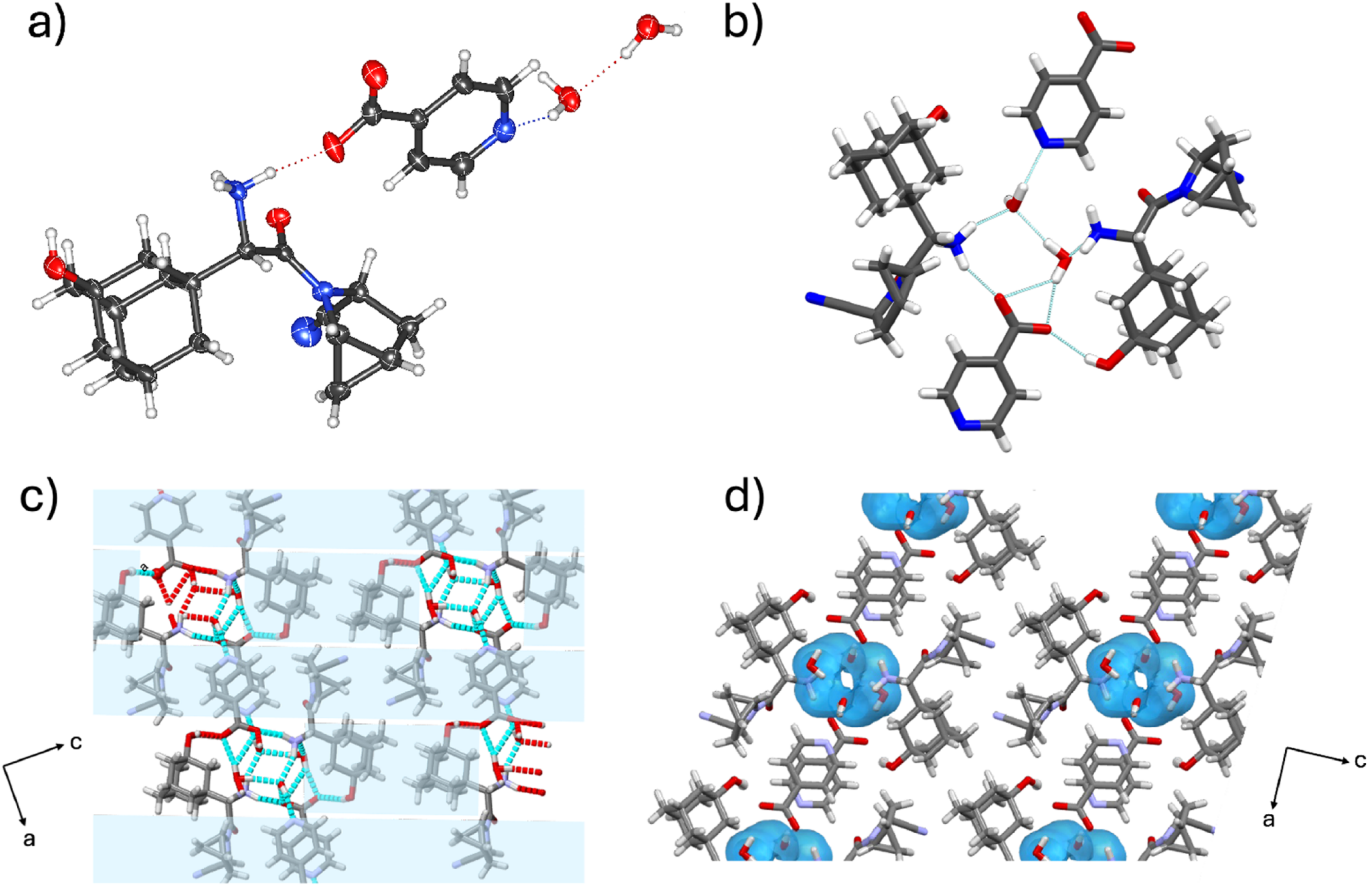
X-ray crystal structure of (**SAX**)·(**INA**)·2­(H_2_O): (a) molecular units connected by [COO^–^···H–^+^NH_2_] charge-assisted hydrogen bonds, (b) hydrogen-bonded array of neighboring **SAX**, **INA** and water units, (c) lipophilic assemblies and hydrophilic pockets of **SAX** aggregates in the *ac*-plane, and (d) calculated water space in the *ac*-plane.

### 3.3 Thermal Gravimetric Analysis of Multicomponent Solids of SAX

SCXRD analysis demonstrated the propensity of **SAX** to form salts with varying levels of hydration. Notably, it is estimated that one in every three APIs can exist in hydrated form.^71,72^ Hydration has been shown to influence the stability, solubility, and bioavailability of APIs^73–75^ often enabling the development of viable pharmaceutical formulations.^76^

TGA analysis was performed using a 10 ^°^C/min heating rate to assess the thermal behavior of the synthesized **SAX** salts and commercial formulations, and to examine the impact of water content on solid-state stability (Figure 7). Dehydration temperature ranges were determined by periods of significant weight loss (weight change threshold: 0.05%). The TGA trace of the **SAX** free base (Combi-Blocks) showed a 2.8% weight loss at 88–138 ^°^C, consistent with the dehydration of the hemihydrate form of **SAX** (2.8% calc.)^34^ Among the synthesized solids, (**SAX**)·(**SUC**) showed minimal weight loss (<2%) below 175 ^°^C, after which rapid decomposition occurred. In contrast, 2­(**SAX**)·(**ADI**)·6­(H_2_O) displayed a sharp weight loss of 12.2% between 40 and 140 ^°^C, in agreement with the expected mass loss from dehydration (12.6%). The 2­(**SAX**)·2­(**SAL**)·(H_2_O) salt showed an initial 1.8% weight loss between 50 and 108 ^°^C, consistent with the predicted value (1.9%) for dehydration. The solid 2­(**SAX**)·2­(**SSA**)·2­(H_2_O) exhibited a gradual 5.5% weight loss up to 190 ^°^C, which is higher than its theoretical value (3.3%). The difference is attributed to partial decomposition of **SAX** at higher temperatures. The salt (**SAX**)·(**INA**)·2­(H_2_O) showed a 7.9% weight loss between 40 and 140 ^°^C, again correlating with the expected dehydration (7.6%). At temperatures exceeding 200 ^°^C, all **SAX**-based solids displayed a significant weight loss, consistent with thermal decomposition processes.

**Figure 7:**
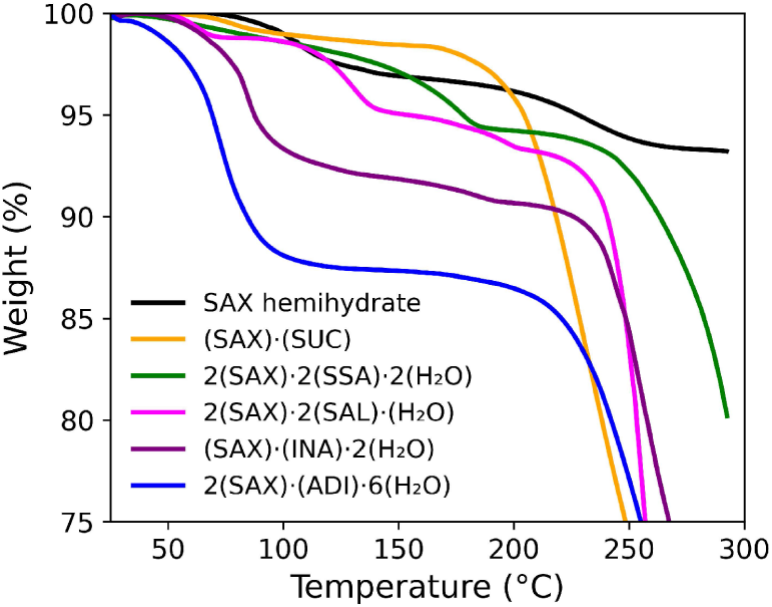
TGA traces of **SAX** solids (10^°^/min ramp rate).

## 4 Conclusions

In summary, we have synthesized five new multicomponent salts of the antidiabetic drug **SAX** with pharmacologically acceptable carboxylic acids. Specifically, salts formed with **SUC**, **ADI**, **SAL**, **SSA**, and **INA** demonstrated the consistent ability of the primary amine in **SAX** to act as a hydrogen acceptor for carboxylic acids. All structures exhibit charge-assisted [COO^–^···H–^+^NH_2_] hydrogen bonding as the primary supramolecular interaction. Salt formation in each case aligns with predictions based on the p*K*_a_ rule.

Notably, **SAX** adopts varying hydration levels across the series, ranging from the anhydrous form in (**SAX**)·(**SUC**) to the hexahydrated form 2­(**SAX**)·(**ADI**)·6­(H_2_O). The differences appear to arise from packing efficiency and hydrogen-bonding requirements in the crystal lattice. The (**SAX**)·(**SUC**) salt is efficiently close-packed, minimizing the need for water. In contrast, larger, bulkier and flexible acids (e.g., **ADI**, **SSA**) likely create larger interstitial voids and additional hydrogen-bonding sites. In these cases, water molecules are incorporated into the lattice to act as ″structural glues″, as observed in similar cocrystal and salt hydrates.^47,77,78^ DFT calculations and electrostatic potential (ESP) maps showed the formation of multiple hydrogen-bond acceptor sites on both **SAX** and the salt-formers, thereby facilitating water inclusion.

Thermogravimetric analysis showed hydration level to influence thermal stability, with (**SAX**)·(**SUC**) and 2­(**SAX**)·2­(**SSA**)·2­(H_2_O) exhibiting stabilities comparable to commercially available **SAX**. The results suggest that salt formation can serve as an effective strategy to modulate the stability of **SAX** toward the development of solid or liquid formulations.^76^

Given the projected global increase in diabetes prevalence to 12.2% by 2045,^79^ we envisage that the strategy described here could inform the design of novel multicomponent solid formulations of **SAX** with improved pharmaceutical properties and potential for expedited FDA approval. We are currently exploring the use of additional salt-formers to generate formulations of **SAX** and related diabetes medications with varying stoichiometries and modulated pharmacokinetic properties.^80^

## Supplementary Material













## Notes

Experimental information, FT-IR and NMR spectra, PXRD patterns, and additional SCXRD data (PDF)
X-ray crystallographic information files (CIF) for (**SAX**)·(**SUC**) (CIF)
X-ray crystallographic information files (CIF) for 2­(**SAX**)·2­(**SAL**)·(H_2_O) (CIF)
X-ray crystallographic information files (CIF) for 2­(**SAX**)·2­(**SSA**)·2­(H_2_O) (CIF)
X-ray crystallographic information files (CIF) for (**SAX**)·(**INA**)·2­(H_2_O) (CIF)
X-ray crystallographic information files (CIF) for 2­(**SAX**)·(**ADI**)·6­(H_2_O) (CIF)

## #

Department of Pharmaceutical Sciences, University of Michigan College of Pharmacy, 1007 E. Huron St., Ann Arbor, MI 48104, United States

## ⊥

Department of Chemistry, Northwestern University, 2145 Sheridan Road, Evanston, Illinois 60208, United States.
