# Supplementary material for: Multicomponent Salts of the Antidiabetic Agent Saxagliptin: Hydration-Assisted Assembly and Lipophilic Aggregation with Carboxylic Acids
Source: ACS Omega. 2026 Mar 17;11(12):19682–91. doi: 10.1021/acsomega.5c13537 (PMC13044837; doi:10.1021/acsomega.5c13537)
Supplement: Supplementary file 1 [file ao5c13537_si_001.pdf]

Supporting Information (SI)

Multicomponent Salts of the Antidiabetic Agent Saxagliptin: Hydration-Assisted Assembly and Lipophilic Aggregation with Carboxylic Acids

Alice E. Colatrella,[†,§] Vanshika Bhaniramka,[†,§] Peter F. Gilbert,[†] Josephine Bicknell,[‡] Maria Pascual-Pedro,[¶] and Gonzalo Campillo-Alvarado[*,†]

[†]Department of Chemistry, Reed College, 3203 SE Woodstock Blvd, Portland, OR 97202-8199, United States, [‡]Department of Chemistry, Georgetown University, 37th and O Streets NW, Washington, District of Columbia 20057-1227, United States. [¶]School of Public Health, Oregon Health & Science University-Portland State University School of Public Health, 1810 SW 5th Avenue, Suite 510, Portland, OR, 97201, United States.

[§]These authors contributed equally to this work.

*e-mail: gcampillo@reed.edu

Table of Contents:

S1) Experimental information
S2) Single-crystal X-ray diffraction data
S3) Powder X-ray diffraction data
S4) FT-IR spectroscopy data
S5) NMR spectroscopy data

S1. Experimental information

Materials:

Methanol and acetonitrile were purchased from Sigma-Aldrich. Compounds succinic acid (**SUC**), salicylic acid (**SAL**), 5-sulfosalicylic acid (**SSA**), isonicotinic acid (**ISO**) and adipic acid (**ADI**) were purchased from AmBeed, and saxagliptin (**SAX**) was purchased from Combi-Blocks. All chemicals were used as received without further purification.

Single crystals of hydrates of **SAX** and (**SAX**)•(**HCl**) were afforded using previously reported methods.[1,2] Crystallization of **SAX** hydrate: Saxagliptin (**SAX,** 31.5 mg, 0.1 mmol**)** was dissolved in 3 mL of methanol. The solution was left for one week of slow crystallization, and suitable crystals for single crystal X-ray diffraction (SCXRD) were collected. The amounts for the components used in salt formation were determined by a preliminary crystallization screening using a 1:1 ratio of **SAX** and the salt former.

Synthesis of (**SAX**)•(**SUC**): Saxagliptin (**SAX**, 39.4 mg, 0.125 mmol) and succinic acid (**SUC**, 14.8 mg, 0.125 mmol) were dissolved in 2 mL of acetonitrile. The sample was heated until the solution was completely transparent. The solution was left for one week of slow crystallization, and colorless, nucleated crystals suitable for SCXRD were collected.

Synthesis of 2(**SAX**)•(**ADI**)•6(H_2O): Saxagliptin (**SAX,** 39.4 mg, 0.125 mmol) and adipic acid (**ADI**, 18.3 mg, 0.065 mmol) were dissolved in 3 mL of methanol. The sample was heated until the solution was completely transparent. The solution was left for one week of slow crystallization, and colorless ageometric crystals suitable for SCXRD were collected.

Synthesis of 2(**SAX**)•2(**SAL**)•(H_2O): Saxagliptin (**SAX,** 39.4 mg, 0.125 mmol) and salicylic acid (**SAL**, 17.3 mg, 0.125 mmol) were dissolved in 3 mL of methanol. The sample was heated until the solution was completely transparent. The solution was left to slowly crystallize for one week, and colorless, needle-like crystals suitable for SCXRD were collected.

Synthesis of 2(**SAX**)•2(**SSA**)•2(H_2O): Saxagliptin (**SAX,** 39.4 mg, 0.125 mmol) and 5-sulfosalicylic acid (**SSA**, 127.3 mg, 0.125 mmol) were dissolved in 3 mL of methanol. The sample was heated until the solution was completely transparent. The solution was left for one week of slow crystallization, and colorless, agglomerated crystals suitable for SCXRD were collected.

Synthesis of (**SAX**)•(**INA**)•2(H_2O): Saxagliptin (**SAX,** 39.4 mg, 0.125 mmol) and isonicotinic acid (**INA**, 15.4 mg, 0.125 mmol) were dissolved in 3 mL of methanol. The sample was heated until the solution was completely transparent. The solution was left for one week of slow crystallization, and colorless, needle-like crystals suitable for SCXRD were collected.

Computational methods:

The molecular structures of the starting materials (**SAX**, **SUC**, **ADI**, **SAL**, **SSA**, and **INA**) were built using *Avogadro* (v1.2.0), and their geometries were initially preoptimized with the Universal Force Field (UFF). These pre-optimized coordinates were then subjected to full geometry optimizations at the ground state level using density functional theory (DFT) with the B3LYP functional and the 6-31G(d,p) basis set, as implemented in *Gaussian 09W*. The resulting optimized geometries were subsequently employed to compute electrostatic potential (ESP) maps via single-point energy calculations at the same level of theory.

S2. Single-crystal X-ray data

Table S1. Crystallographic parameters for 2(**SAX**)•(**ADI**)•6(H_2O)

Compound name	2(**SAX**)•(**ADI**)•6(H_2O)
Empirical formula	$C_{21}H_{36}N_3O_7$
Formula weight	442.53
Temperature/K	300(12)
Crystal system	monoclinic
Space group	*I*2
a/Å	11.3042(11)
b/Å	6.7846(6)
c/Å	30.642(3)
α/°	90
β/°	98.334(9)
γ/°	90
Volume/Å^3	2325.3(4)
Z	4
ρ_{calc}g/cm^3	1.264
μ/mm^{-1}	0.095
F(000)	956.0
Crystal size/mm^3	0.7 × 0.121 × 0.092
Radiation	Mo Kα (λ = 0.71073)
2Θ range for data collection/°	5.026 to 49.998
Index ranges	-12 ≤ h ≤ 13, -8 ≤ k ≤ 7, -35 ≤ l ≤ 36
Reflections collected	8011
Independent reflections	3878 [R_{int} = 0.0554, R_{sigma} = 0.0693]
Data/restraints/parameters	3878/10/300
Goodness-of-fit on F^2	0.932
Final R indexes [I>=2σ (I)]	R_1 = 0.0450, wR_2 = 0.0996
Final R indexes [all data]	R_1 = 0.0658, wR_2 = 0.1084
CDCC Identification Code	2475448

Table S2. Crystallographic parameters for (**SAX**)•(**INA**)•2(H_2O)

Compound name	(**SAX**)•(**INA**)•2(H_2O)
Empirical formula	$C_{24}H_{34}N_4O_6$
Formula weight	474.55
Temperature/K	182(3)
Crystal system	monoclinic
Space group	$P2_1$
a/Å	11.1468(9)
b/Å	6.7985(6)
c/Å	16.4426(14)
α/°	90
β/°	109.743(9)
γ/°	90
Volume/Å^3	1172.80(18)
Z	2
ρ_{calc}g/cm^3	1.344
μ/mm^{-1}	0.097
F(000)	508.0
Crystal size/mm^3	0.464 × 0.087 × 0.055
Radiation	Mo Kα (λ = 0.71073)
2Θ range for data collection/°	5.376 to 50.992
Index ranges	$-13 \leq h \leq 13$, $-8 \leq k \leq 8$, $-18 \leq l \leq 19$
Reflections collected	8919
Independent reflections	3894 [R_{int} = 0.0365, R_{sigma} = 0.0584]
Data/restraints/parameters	3894/7/321
Goodness-of-fit on F^2	0.995
Final R indexes [I>=2σ (I)]	R_1 = 0.0433, wR_2 = 0.0869
Final R indexes [all data]	R_1 = 0.0637, wR_2 = 0.0937
CDCC Identification Code	2475452

Table S3. Crystallographic parameters for 2(**SAX**)•2(**SSA**)•2(H_2O)

Compound name	2(**SAX**)•2(**SSA**)•2(H_2O)
Empirical formula	$C_{50}H_{65}N_6O_{18}S_2$
Formula weight	1102.20
Temperature/K	294(3)
Crystal system	monoclinic
Space group	$P2_1$
a/Å	6.8091(3)
b/Å	33.7219(16)
c/Å	11.5595(5)
α/°	90
β/°	94.469(4)
γ/°	90
Volume/Å^3	2646.2(2)
Z	2
ρ_{calc}g/cm^3	1.383
μ/mm^{-1}	0.180
F(000)	1166.0
Crystal size/mm^3	0.572 × 0.143 × 0.117
Radiation	Mo Kα (λ = 0.71073)
2Θ range for data collection/°	4.282 to 61.33
Index ranges	$-9 \leq h \leq 8$, $-42 \leq k \leq 47$, $-15 \leq l \leq 16$
Reflections collected	25931
Independent reflections	12761 [R_{int} = 0.0311, R_{sigma} = 0.0615]
Data/restraints/parameters	12761/63/742
Goodness-of-fit on F^2	0.999
Final R indexes [I>=2σ (I)]	R_1 = 0.0564, wR_2 = 0.1269
Final R indexes [all data]	R_1 = 0.0889, wR_2 = 0.1427
CDCC Identification Code	2475451

Table S4. Crystallographic parameters for (**SAX**)•(**SUC**)

Compound name	(**SAX**)•(**SUC**)
Empirical formula	$C_{22}H_{31}N_3O_6$
Formula weight	433.50
Temperature/K	296.7(3)
Crystal system	monoclinic
Space group	$P2_1$
a/Å	11.6031(10)
b/Å	6.8584(5)
c/Å	13.4382(10)
α/°	90
β/°	101.846(8)
γ/°	90
Volume/Å^3	1046.62(14)
Z	2
ρ_{calc}g/cm^3	1.376
μ/mm^{-1}	0.101
F(000)	464.0
Crystal size/mm^3	0.934 × 0.739 × 0.323
Radiation	Mo Kα (λ = 0.71073)
2Θ range for data collection/°	5.198 to 51.996
Index ranges	-14 ≤ h ≤ 14, -8 ≤ k ≤ 8, -16 ≤ l ≤ 16
Reflections collected	6763
Independent reflections	3504 [R_{int} = 0.0622, R_{sigma} = 0.0673]
Data/restraints/parameters	3504/63/342
Goodness-of-fit on F^2	1.027
Final R indexes [I>=2σ (I)]	R_1 = 0.0512, wR_2 = 0.1315
Final R indexes [all data]	R_1 = 0.0639, wR_2 = 0.1395
CDCC Identification Code	2475449

Table S5. Crystallographic parameters for 2(**SAX**)•2(**SAL**)•(H_2O)

Compound name	2(**SAX**)•2(**SAL**)•(H_2O)
Empirical formula	$C_{50}H_{64}N_6O_{11}$
Formula weight	925.07
Temperature/K	102.3(8)
Crystal system	monoclinic
Space group	$P2_1$
a/Å	13.724(2)
b/Å	6.7104(10)
c/Å	25.454(4)
α/°	90
β/°	92.290(16)
γ/°	90
Volume/Å^3	2342.3(7)
Z	2
ρ_{calc}g/cm^3	1.312
μ/mm^{-1}	0.093
F(000)	988.0
Crystal size/mm^3	0.744 × 0.069 × 0.061
Radiation	Mo Kα (λ = 0.71073)
2Θ range for data collection/°	4.28 to 50
Index ranges	$-16 \le h \le 15$, $-7 \le k \le 7$, $-30 \le l \le 30$
Reflections collected	11947
Independent reflections	7039 [R_{int} = 0.1511, R_{sigma} = 0.2855]
Data/restraints/parameters	7039/1/607
Goodness-of-fit on F^2	0.809
Final R indexes [I>=2σ (I)]	R_1 = 0.0814, wR_2 = 0.1530
Final R indexes [all data]	R_1 = 0.2113, wR_2 = 0.1992
CDCC Identification Code	2475450

Table S6. Selected hydrogen bonds and interactions in **SAX** salts.

**crystal/ parameter**	***Interaction type (just for reference)***	***d*(C-H···O) (Å)**	***d*(N-H···O) (Å)**	***d*(N···H-O) (Å)**	***d*(C-H···N) (Å)**	***d*(O-H···O) (Å)**	***d*(O-H···S) (Å)**	***d*(N-H···S) (Å)**	***d*(N-H···N) (Å)**	**symmetry code**
2(**SAX**)•(**ADI**)•6(H_2O)	[1]C2-H2···O6	[1]3.480(5)	-		-					-
	[2]C24-H24···O6	[2]3.613(5)	-		-					-
	[3]C21-H21···O2	[3]3.081(4)	-		-					(-½+X,-½+Y,½+Z)
	[4]C23-H23···O1	[4]3.187(5)	-		-					(½+X,½+Y,½+Z)
	[5]N3-H3···O4									-
	[6]N3-H3···O4*		[6]2.764(14)							(1-X, +Y, 1-Z)
	[7]C11-H11···N1				[7]3.57(3)					(½-X, -½+Y,½, ½-Z)
	[8]C14-H14···O7	[8]3.758(6)								-
	[9]O5-H5···O4					[9]2.809(4)				(1-X, +Y, 1-Z)
	[10]O5-H5···O3					[10]2.769(5)				(1-X, +Y, 1-Z)
	[11]O2-H2···O7					[11]2.798(5)				(+X, -1+Y, +Z)
	[12]O7-H7···O5					[12]2.850(5)				(1-X, 1+Y, 1-Z)
	[13]O6-H6···O3					[8]2.829(5)				-
	[14]C4-H4···O6	[14]3.764(5)								(+X, 1+Y,+Z)
	[15]N3-H3···O6		[15]2.871(5)							(1-X, +Y, 1-Z)
	[16]O6-H6···O3*					[16]2.835(4)				(1-X, +Y, 1-Z)
	[17]C23-H23···O7	[17]4.17(6)								(1+X, +Y, +Z)
	[18]C22-H22···O7	[28]4.08(6)								(1+X, +Y, +Z)
	[19]C8-H8···O7	[19]3.81(4)								(+X, 1+Y, +Z)
	[20]C9-H9···N1				[20]3.92(4)					(½-X, -½+Y, ½-Z)
	[21]C26-H26···N1				[21]3.98(4)					-
	[22]C4-H4···N1				[22]4.96(3)					-
	[23]C6-H6···O6	[23]4.50(2)								-
	[24]C13-H13···O7	[24]3.95(4)								(2-X, +Y, 1-Z)
	[25]C13-H13···O7	[25]4.11(4)								(2-X, -1+Y, 1-Z)
	[26]C13-H13···O1	[26]4.055(15)								(1-X, +Y, 1-Z)
	[27]C14-H14···O2	[27]3.895(17)								-

**crystal/ parameter**	***Interaction type (just for reference)***	***d*(C-H···O) (Å)**	***d*(N-H···O) (Å)**	***d*(N···H-O) (Å)**	***d*(C-H···N) (Å)**	***d*(O-H···O) (Å)**	***d*(O-H···S) (Å)**	***d*(N-H···S) (Å)**	***d*(N-H···N) (Å)**	**symmetry code**
**(SAX)•(INA)•** 2(H_2O)	[28]O1-H1···O4					[28]2.996(4)				(2-X, ½+Y, 1-Z)
	[29]C17-H17···O1	[29]3.222(4)								(1+X, +Y, +Z)
	[30]C22-H22···O1	[30]3.417(4)								(1+X, +Y, +Z)
	[31]C24-H24···O1	[31]3.791(4)								(2-X, ½+Y, 1-Z)
	[32]C21-H21···O2	[32]3.951(4)								-
	[33]C23-H23···O2	[33]3.184(4)								(1-X, ½+Y, 1-Z)
	[34]C15-H15···O2	[34]3.191(4)								(+X, -1+Y, +Z)
	[35]N1-H1···O3		[35]2.727(4)							-
	[36]O5-H5···O3					[36]2.756(4)				(1-X, -½+Y, 1-Z)
	[37]O6-H6···O3					[37]3.359(4)				(-1+X, +Y, +Z)
	[38]N1-H1···O4		[38]4.746(3)							-
	[39]O6-H6···O4					[39]2.903(4)				(-1+X, +Y, +Z)
	[40]C11-H11···O4	[40]3.415(4)								(2-X, -½+Y, 1-Z)
	[41]C8-H8···O4	[41]3.705(4)								(2-X, -½+Y, 1-Z)
	[42]O6-H6···O5					[42]2.788(4)				-
	[43]N4···H5-O5			[43]2.737 (4)						-
	[44]O5-H5···O4					[44]3.538(4)				(1-X, -½+Y, 1-Z)
	[45]N1-H1···O5,		[45]2.742(3)							(-1+X, +Y, +Z)
	[46]N1-H1···O6		[46]2.930(4)							(1-X, -½+Y, 1-Z)
	[47]C11-H11···O6	[47]3.647(4)								(1-X, -½+Y, 1-Z)
	[48]C12-H12···O6	[49]3.626(4)								(1-X, -½+Y, 1-Z)
	[50]C2-H2···O6	[50]3.515(4)								(1-X, -½+Y, 1-Z)
	[51]C4-H4···O6	[51]3.827(4)								(1-X, -½+Y, 1-Z)
	[52]C21-H21···N1				[52]4.599(4)					-
	[53]C21-H21···N2				[53]4.011(4)					-
	[54]C12-H12···N3				[54]4.645(5)					-
	[55]C14-H14···N3				[55]3.979(6)					-
	[56]C7-H7···N3				[56]3.834(5)					(2-X, -½+Y, 2-Z)
	[57]C4-H4···N3				[57]3.736(5)					(2-X, -½+Y, 2-Z)
	[58]C5-H5···N3				[58]3.611(5)					(2-X, -½+Y, 2-Z)
	[59]C6-H6···N3				[59]3.809(6)					(2-X, -½+Y, 2-Z)

**crystal/ parameter**	***Interaction type (just for reference)***	***d*(C-H···O) (Å)**	***d*(N-H···O) (Å)**	***d*(N··· H-O) (Å)**	***d*(C-H···N) (Å)**	***d*(O-H···O) (Å)**	***d*(O-H···S) (Å)**	***d*(N-H···S) (Å)**	***d*(N-H···N) (Å)**	**symmetry code**
	[60]N4···H6-O6			[60]4.463 (6)						-
2(**SAX**)•2(**SSA**)•2(H_2O)	[61]C10-H10···O1	[61]3.618(13)								(1+X, +Y, +Z)
	[62]C42-H42···O1	[61]3.788(12)								(1-X, -½+Y, 2-Z)
	[63]C43-H43···O1	[63]3.494(11)								(1-X, -½+Y, 2-Z)
	[64]C40-H40···O1	[64]4.0225(11)								(1-X, -½+Y, 2-Z)
	[65]N6-H6···O1		[65]2.190(12)							(1+X, +Y, +Z)
	[66]C14-H14···O2	[66]3.264(6)								(-1+X, +Y, +Z)
	[67]O14-H14···O2					[67]3.111(5)				(+X, +Y, 1+Z)
	[68]O3-H3···O12					[68]2.624(4)				-
	[69]N4-H4···O3		[69]4.703(4)							-
	[70]N1-H1···O3		[70]2.792(4)							-
	[71]O4-H4···O16					[71]2.806(5)				-
	[72]N1-H1···O4		[72]3.242(5)							-
	[73]N4-H4···O4		[73]3.639(5)							-
	[74]C48-H48···O4	[74]4.576(6)								-
	[75]N1-H1···O5		[75]2.837(4)							-
	[76]N4-H4···O5									-
	[77]O16-H16···O5					[76]3.559(4)				-
	[78]C27-H27···O6	[78]3.351(5)								-
	[79]O11-H11···O6					[79]2.611(4)				-
	[80]O6-H6···S1						[80]3.504(3)			-
	[81]C46-H46···O7	[81]4.848(6)								-
	[82]O8-H8···O7					[82]2.624(5)				-
	[83]O6-H6···O16					[83]4.348(4)				(+X, +Y, -1+Z)
	[84]O7-H7···O16					[84]2.855(5)				(+X, +Y, -1+Z)
	[85]C46-H46···O8	[85]4.536(6)								-
	[86]C47-H47···O8	[86]4.612(6)								-
	[87]C36-H36···O8	[87]3.547(5)								(-1+X, +Y, -1+Z)
	[88]O17-H17···O8					[88]2.907(6)				(-1+X, +Y, -1+Z)
	[89]O15-H15···O9					[89]2.775(5)				-

**crystal/ parameter**	***Interaction type (just for reference)***	***d*(C-H···O) (Å)**	***d*(N-H···O) (Å)**	***d*(N···H-O) (Å)**	***d*(C-H···N) (Å)**	***d*(O-H···O) (Å)**	***d*(O-H···S) (Å)**	***d*(N-H···S) (Å)**	***d*(N-H···N) (Å)**	**symmetry code**
	[90]O15-H15···O10					[90]4.305(5)				-
	[91]N1-H1···O10		[91]2.818(5)							(+X, +Y, -1+Z)
	[92]C30-H30···O10	[92]3.464(6)								(-1+X, +Y, +Z)
	[93]C31-H31···O10	[93]3.490(6)								(-1+X, +Y, +Z)
	[94]C4A-H4A···O10	[94]4.258(19)								(+X, +Y, -1+Z)
	[95]C6-H6···O10	[95]3.206(8)								(+X, +Y, -1+Z)
	[96]N4-H4···O10		[96]4.562(5)							(+X, +Y, -1+Z)
	[97]N4-H4···O11		[97]2.832(4)							(+X, +Y, -1+Z)
	[98]C36-H36···O11	[98]3.312(5)								(+X, +Y, -1+Z)
	[99]C20-H20···O12	[99]3.336(6)								-
	[100]C15-H15···O12	[100]4.110(7)								-
	[102]O14-H14···O13					[102]2.657(5)				-
	[103]O15-H15···O13					[103]2.875(5)				(+X, +Y, 1+Z)
	[104]O15-H15···O12					[104]4.391(5)				(+X, +Y, 1+Z)
	[105]C14-H14···O14	[105]4.244(7)								-
	[106]C16-H16···O14	[106]3.417(6)								(1+X, +Y, +Z)
	[107]C31-H31···O15	[107]5.043(6)								-
	[108]O15-H15···S1						[108]3.914(5)			-
	[109]C4-H4···O15	[109]3.790(8)								(1+X, +Y, -1+Z)
	[110]C6-H6···O15	[110]4.459(8)								(1+X, +Y, -1+Z)
	[111]C24-H24···O16	[111]4.031(6)								-
	[112]C48-H48···O16	[112]3.782(5)								-
	[113]C36-H36···O16	[113]3.961(5)								-
	[114]C37-H37···O16	[114]4.157(4)								(+X, +Y, 1+Z)
	[115]C34-H34···O16	[115]3.473(5)								-
	[116]O16-H16···S2						[116]3.797(4)			-
	[117]C47-H47···O18	[117]3.185(5)								(1+X, +Y, +Z)
	[118]N1-H1···S2							[118]3.593(4)		-
	[119]N1-H1···O15		[119]2.847(5)							-
	[120]N1-H1···S1							[120]3.821(4)		(+X, +Y, 1+Z)
	[121]C5A-H5A···N3				[120]4.319(17)					-
	[122]C4-H4···N3				[121]4.745(10)					-
	[123]C18-H18···N3				[122]3.872(8)					-

crystal/ parameter	*Interaction type (just for reference)*	***d*(C-H···O) (Å)**	***d*(N-H···O) (Å)**	***d*(N···H-O) (Å)**	***d*(C-H···N) (Å)**	***d*(O-H···O) (Å)**	***d*(O-H···S) (Å)**	***d*(N-H···S) (Å)**	***d*(N-H···N) (Å)**	**symmetry code**
	[123]C41-H41···N3				[122]3.440(8)					(-X, -½+Y, 1-Z)
	[124]N4-H4···O16		[124]2.873(5)							-
	[125]N4-H4···S1							[125]3.587(4)		(+X, +Y, 1+Z)
	[126]C5-H5···N6				[126]3.996(9)					(1-X, 1/2+Y, 1-Z)
	[127]C10-H10···N6				[127]3.831(11)					(1-X, 1/2+Y, 1-Z)
	[128]C11A-H11A···N6				[128]3.79(2)					(1-X, 1/2+Y, 1-Z)
	[129]C49-H49···N6				[129]4.044(8)					-
**(SAX)•(SUC)**	[130]O1-H1···O5					[130]2.942(6)				(-1-X, -½+Y, -1-Z)
	[131]C8-H8···O1	[131]3.397(12)								(+X, 2+Y, +Z)
	[132]C21-H21···O1	[132]3.373(7)								(-1-X, 3/2+Y, -1-Z)
	[133]N1-H1···O3		[133]300(4)							-
	[134]N1-H1···O3*		[134]2.788(4)							(-1-X, ½+Y, -1-Z)
	[135]C4-H4···O3	[135]4.041(13)								(+X, 1+Y, +Z)
	[136]C9-H9···O3	[136]3.542(10)								-
	[137]N1-H1···O4		[137]3.624(4)							(-1-X, ½+Y, -1-Z)
	[138]N1-H1···O4*		[138]2.947(4)							-
	[139]C4-H4···O4	[139]3.587(14)								(-1-X, ½+Y, -1-Z)
	[140]C17-H17···O4	[140]4.257(5)								(+X, 1+Y, +Z)
	[141]C13-H13···O4	[141]3.679(5)								-
	[142]C15-H15···O5	[142]3.284(6)								(-2-X, -½+Y, -1-Z)
	[143]C12-H12···O5	[143]3.734(11)								(-1-X, -½+Y, -1-Z)
	[144]C20-H20···O6	[144]3.599(5)								(-2-X, -½+Y, -1-Z)
	[145]C21-H21···O6	[145]3.344(5)								(-2-X, -½+Y, -1-Z)
	[146]O6-O6···O4					[146]2.572(4)				(-2-X, -½+Y, -1-Z)
	[147]C17-H17···N1				[147]4.571(5)					(-1-X, -½+Y, -1-Z)
	[148]C9-H9···N3				[148]4.566(11)					(+X, -1+Y, +Z)
	[149]C5-H5···N2				[149]3.305(17)					-
	[150]C5-H5···N3				[150]4.404(18)					-
	[151]C14-H14···N3				[151]3.912(7)					-
	[152]C4-H4···N3				[152]4.702(14)					-

crystal/ parameter	*Interaction type (just for reference)*	***d*(C-H···O) (Å)**	*d*(N-H···O) (Å)	*d*(N···H-O) (Å)	*d*(C-H···N) (Å)	***d*(O-H···O) (Å)**	***d*(O-H···S) (Å)**	***d*(N-H···S) (Å)**	***d*(N-H···N) (Å)**	**symmetry code**
	[153]C7-H7···N3				[153]4.79(2)					-
	[154]C10-H10···N3				[154]4.633(15)					(+X, -1+Y, +Z)
2(**SAX**)•2(**SAL**)•(H_2O)	[155]O1-H1···O11					[155]2.812(12)				-
	[156]O6-H6···O1					[156]2.756(11)				-
	[157]C41-H41···O6	[157]3.636(14)								-
	[158]C5-H5···O2	[158]3.190(13)								-
	[159]C16-H16···O2	[159]4.844(14)								(+X, -1+Y, +Z)
	[160]C2-H2···O2	[160]4.617(14)								(+X, -1+Y, +Z)
	[161]O3-H3···O4					[161]2.506(10)				-
	[162]O3-H3···O2					[162]4.083(11)				(2-X, 3/2+Y, 1-Z)
	[163]C13-H13···O3	[163]3.300(15)								(2-X, 3/2+Y, 1-Z)
	[164]C20-H20···O3	[164]3.668(15)								(2-X, 3/2+Y, 1-Z)
	[165]N1-H1···O4*		[165]2.889(11)							(2-X, 1/2+Y, 1-Z)
	[166]N1-H1···O5		[166]2.798(11)							-
	[167]N1-H1···O5*		[167]3.116(11)							(2-X, 1/2+Y, 1-Z)
	[168]C6-H6···O5	[168]3.514(13)								-
	[169]C4-H4···O6	[169]3.436(12)								-
	[170]C12-H12···O6	[170]3.695(12)								-
	[171]O6-H6···O11					[171]4.447(12)				-
	[172]C27-H27···O7	[172]4.373(13)								(+X, -1+Y, +Z)
	[173]C31-H31···O7	[173]3.015(8)								(+X, -1+Y, +Z)
	[174]O8-H8···O9					[174]2.527(11)				-
	[175]C30-H30···O8	[175]4.170(14)								-
	[176]C30-H30···O9	[176]3.761(13)								-
	[177]N4-H4···O9		[177]2.814(10)							-
	[178]C37-H37···O8	[178]3.690(15)								(+X, 1+Y, +Z)
	[179]C38-H38···O8	[179]3.73917)								(+X, 1+Y, +Z)
	[180]C37-H37···O9	[180]4.008(14)								(+X, 1+Y, +Z)
	[181]C48-H48···O9	[181]4.601(14)								(+X, 1+Y, +Z)
	[182]N4-H4···O10		[182]4.333(10)							-
	[183]N4-H4···O10*		[183]2.781(10)							(1-X, -½+Y, 2-Z)
	[184]N4-H4···O10*		[184]2.911(11)							(+X, -1+Y, +Z)

**crystal/ parameter**	***Interaction type (just for reference)***	***d*(C-H···O) (Å)**	***d*(N-H···O) (Å)**	***d*(N··· H-O) (Å)**	***d*(C-H···N) (Å)**	***d*(O-H···O) (Å)**	***d*(O-H···S) (Å)**	***d*(N-H···S) (Å)**	***d*(N-H···N) (Å)**	**symmetry code**
	[185]C31-H31···O10	[185]4.601(14)								(+X, -1+Y, +Z)
	[186]C10-H10···O11	[186]3.525(4)								-
	[187]C60-H60···O11	[187]4.479(15)								-
	[188]C4-H4···O11	[188]3.624(14)								-
	[189]C4-H4···O11*	[189]3.283(10)								(+X, -1+Y, +Z)
	[190]N3··H11-O11	[190]2.939(17)								-
	[191]N6··H11-O11	[191]3.664(18)								-
	[192]C6-H6···O11	[192]4.039(14)								(+X, -1+Y, +Z)
	[192]C17-H17···O11	[192]4.257(15)								(+X, -1+Y, +Z)
	[193]C12-H12···O11	[193]3.738(4)								(+X, -1+Y, +Z)
	[194]C20-H20···N1				[194]4.805(15)					-
	[195]C17-H17···N3				[195]3.602(16)					-
	[196]C17-H17···N3*				[196]3.188(15)					(+X, -1+Y, +Z)
	[197]C4-H4···N3				[197]4.135(14)					-
	[198]C16-H16···N3				[198]3.196(16)					(+X, -1+Y, +Z)
	[199]N4-H4···N5								[199]4.540(11)	(1-X, ½+Y, 2-Z)
	[200]C16-H16···N4				[200]4.554(13)					(1-X, ½+Y, 2-Z)
	[201]C41-H41···N6				[201]3.802(17)					-
	[202]C29-H29···N6				[202]4.535(15)					-

[1]C2-H2···O6, [2]C24-H24···O6, [3]C21-H21···O2, [4]C23-H23···O1, [5]N3-H3···O4, [6]N3-H3···O4*, [7]C11-H11···N1, [8]C14-H14···O7, [9]O5-H5···O4, [10]O5-H5···O3, [11]O2-H2···O7, [12]O7-H7···O5, [13]O6-H6···O3, [14]C4-H4···O6, [15]N3-H3···O6, [16]O6-H6···O3*, [17]C23-H23···O7, [18]C22-H22···O7, [19]C8-H8···O7, [20]C9-H9···N1, [21]C26-H26···N1,[22]C4-H4···N1, [23]C6-H6···O6, [24]C13-H13···O7, [25]C13-H13···O7, [26]C13-H13···O1, [27]C14-H14···O2

[28]O1-H1···O4,[29]C17-H17···O1,[30]C22-H22···O1,[31]C24-H24···O1,[32]C21-H21···O2,[33]C23-H23···O2,[34]C15-H15···O2,[35]N1-H1···O3,[36]O5-H5···O3,[37]O6-H6···O3,[38]N1-H1···O4,[39]O6-H6···O4,[40]C11-H11···O4,[41]C8-H8···O4,[42]O6-H6···O5,[43]N4···H5-O5,[44]O5-H5···O4,[45]N1-H1···O5,[46]N1-H1···O6,[47]C11-H11···O6,[48]C12-H12···O6,[50]C2-H2···O6,[51]C4-H4···O6,[52]C21-H21···N1,[53]C21-H21···N2,[54]C12-H12···N3,[55]C14-H14···N3,[56]C7-H7···N3,[57]C4-H4···N3,[58]C5-H5···N3,[59]C6-H6···N3,[60]N4···H6-O6

[61]C10-H10···O1,[62]C42-H42···O1,[63]C43-H43···O1,[64]C40-H40···O1,[65]N6-H6···O1,[66]C14-H14···O2,[67]O14-H14···O2,[68]O3-H3···O12,[69]N4-H4···O3,[70]N1-H1···O3,[71]O4-H4···O16,[72]N1-H1···O4,[73]N4-H4···O4,[74]C48-H48···O4,[75]N1-H1···O5,[76]N4-H4···O5,[77]O16-H16···O5,[78]C27-H27···O6,[79]O11-H11···O6,[80]O6-H6···S1,[81]C46-H46···O7,[82]O8-H8···O7,[83]O6-H6···O16,[84]O7-H7···O16,[85]C46-H46···O8,[86]C47-H47···O8,[87]C36-H36···O8,[88]O17-H17···O8,[89]O15-H15···O9,[90]O15-H15···O10,[91]N1-H1···O10,[92]C30-H30···O10,[93]C31-H31···O10,[94]C4A-H4A···O10,[95]C6-H6···O10,[96]N4-H4···O10,[97]N4-H4···O11,[98]C36-H36···O11,[99]C20-H20···O12,[100]C15-H15···O12,[102]O14-H14···O13,[103]O15-H15···O13,

[104]O15-H15···O12,[105]C14-H14···O14,[106]C16-H16···O14,[107]C31-H31···O15,[108]O15-H15···S1,[109]C4-H4···O15,[110]C6-H6···O15,[111]C24-H24···O16,[112]C48-H48···O16,[113]C36-H36···O16,[114]C37-H37···O16,[115]C34-H34···O16,[116]O16-H16···S2,[117]C47-H47···O18,[118]N1-H1···S2,[119]N1-H1···O15,[120]N1-H1···S1,[121]C5A-H5A···N3,[122]C4-H4···N3,[123]C18-H18···N3,[123]C41-H41···N3,[124]N4-H4···O16,[125]N4-H4···S1,[126]C5-H5···N6,[127]C10-H10···N6,[128]C11A-H11A···N6,[129]C49-H49···N6,[130]O1-H1···O5,[131]C8-H8···O1,[132]C21-H21···O1,[133]N1-H1···O3,[134]N1-H1···O3*,[135]C4-H4···O3,[136]C9-H9···O3,[137]N1-H1···O4,[138]N1-H1···O4*,[139]C4-H4···O4,[140]C17-H17···O4,[141]C13-H13···O4,[142]C15-H15···O5,[143]C12-H12···O5,[144]C20-H20···O6,[145]C21-H21···O6,[146]O6-O6···O4,[147]C17-H17···N1,[148]C9-H9···N3[149]C5-H5···N2,

[150]C5-H5···N3,[151]C14-H14···N3,[152]C4-H4···N3,[153]C7-H7···N3,[154]C10-H10···N3,[155]O1-H1···O11,[156]O6-H6···O1,[157]C41-H41···O6,[158]C5-H5···O2,[159]C16-H16···O2,[160]C2-H2···O2,[161]O3-H3···O4,[162]O3-H3···O2,[163]C13-H13···O3,[164]C20-H20···O3,[165]N1-H1···O4*,[166]N1-H1···O5,[167]N1-H1···O5*,[168]C6-H6···O5,[169]C4-H4···O6,[170]C12-H12···O6,[171]O6-H6···O11,[172]C27-H27···O7,[173]C31-H31···O7,[174]O8-H8···O9,[175]C30-H30···O8,[176]C30-H30···O9,

[177]N4-H4···O9,[178]C37-H37···O8,[179]C38-H38···O8,[180]C37-H37···O9,[181]C48-H48···O9,[182]N4-H4···O10,[183]N4-H4···O10*,[184]N4-H4···O10*,[185]C31-H31···O10,[186]C10-H10···O11,[187]C60-H60···O11,[188]C4-H4···O11,[189]C4-H4···O11*,[190]N3··H11-O11,[191]N6··H11-O11,[192]C6-H6···O11,[192]C17-H17···O11,[193]C12-H12···O11,[194]C20-H20···N1,[195]C17-H17···N3,[196]C17-H17···N3*,[197]C4-H4···N3,[198]C16-H16···N3,[199]N4-H4···N5,[200]C16-H16···N4,[201]C41-H41···N6,[202]C29-H29···N6.

Table S6. Root mean square deviation (RMSD) analysis for the conformational overlay of **SAX** units in multicomponent salts.

**Salt**	**RMSD[1] vs. reference**
(**SAX**)·(**SUC**)	0.0 (reference)
2(**SAX**)·(**ADI**)·6(H_2O)	0.2855
2(**SAX**)·2(**SAL**)·(H_2O)	1.5832[2]
2(**SAX**)·2(**SSA**)·2(H_2O)	0.2624
(**SAX**)·(**INA**)·2(H_2O)	0.4001

[1] RMSD values were calculated using CCDC Mercury in Mercury (2025.3.3). [2]Higher RMSD value due to rotation of the adamantanol ring.

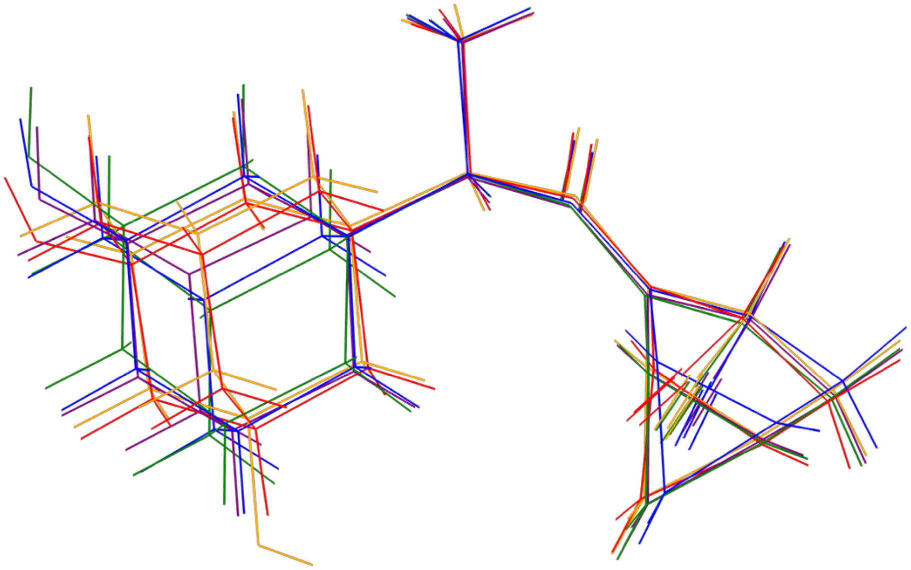

Figure S1. Overlay of **SAX** units in crystal structures of multicomponent salts. Color code: (**SAX**)•(**SUC**) (red), 2(**SAX**)•(**ADI**)•6(H_2O) (blue), 2(**SAX**)•2(**SAL**)•(H_2O) (orange), 2(**SAX**)•2(**SSA**)•2(H_2O) (purple) and (**SAX**)•(**INA**)•2(H_2O) (green).

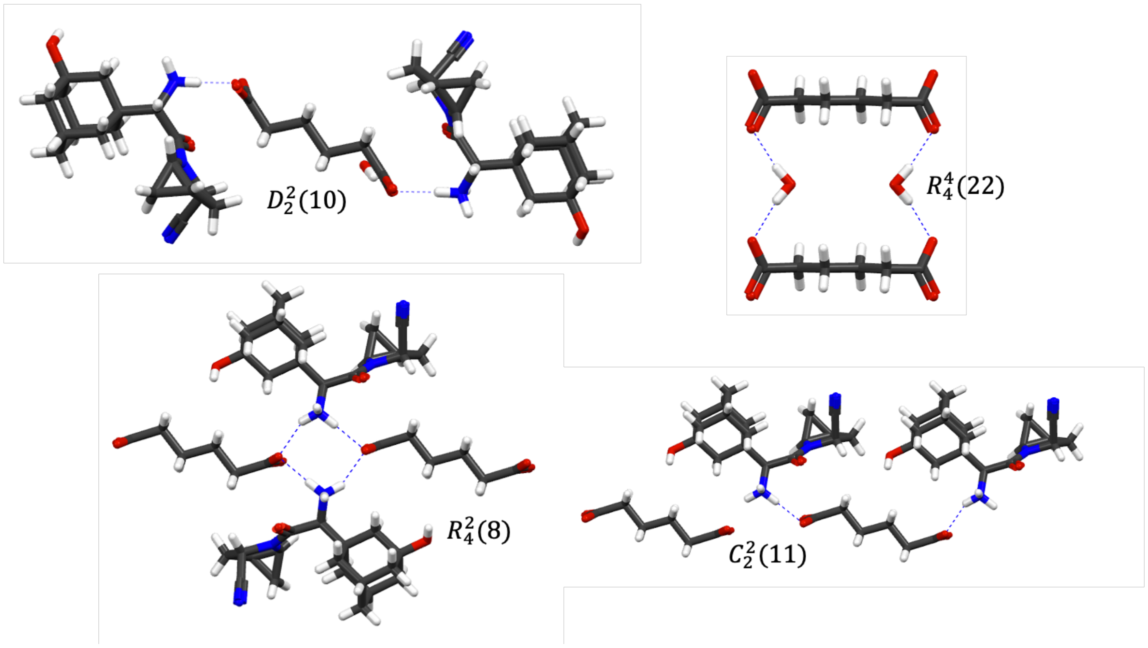

Figure S2. Selected graph sets for 2(**SAX**)•(**ADI**)•6(H_2O).

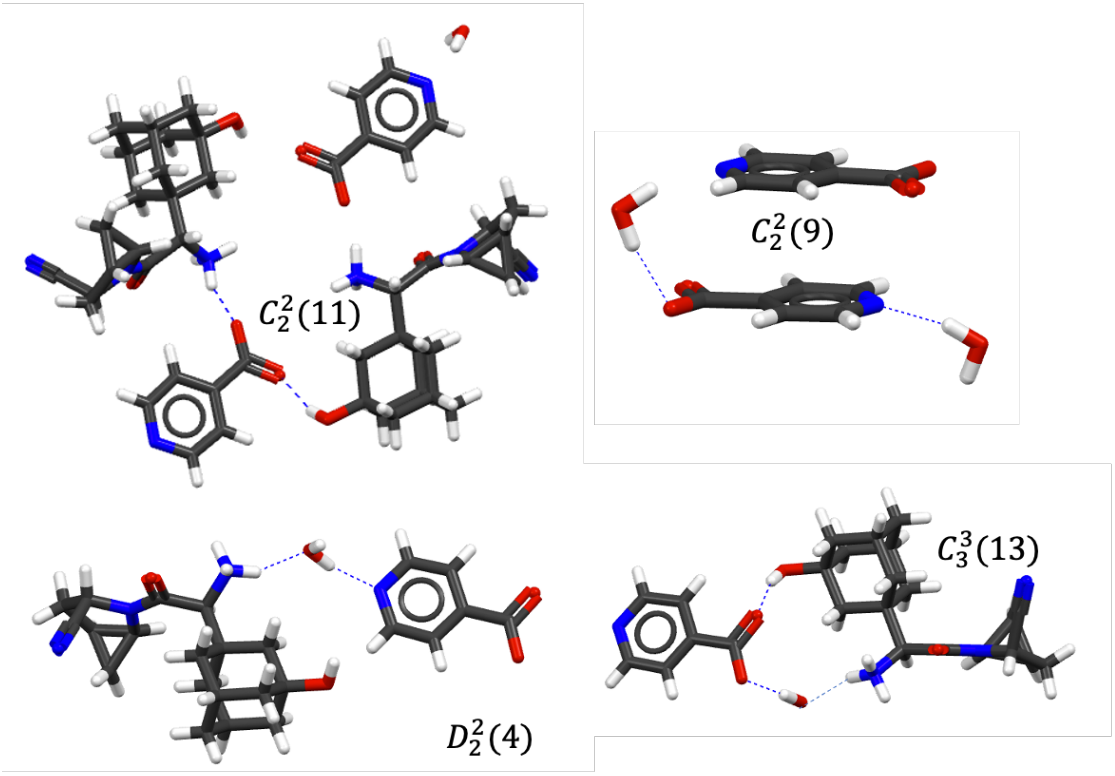

Figure S3. Selected graph sets for (**SAX**)•(**INA**)•2(H_2O).

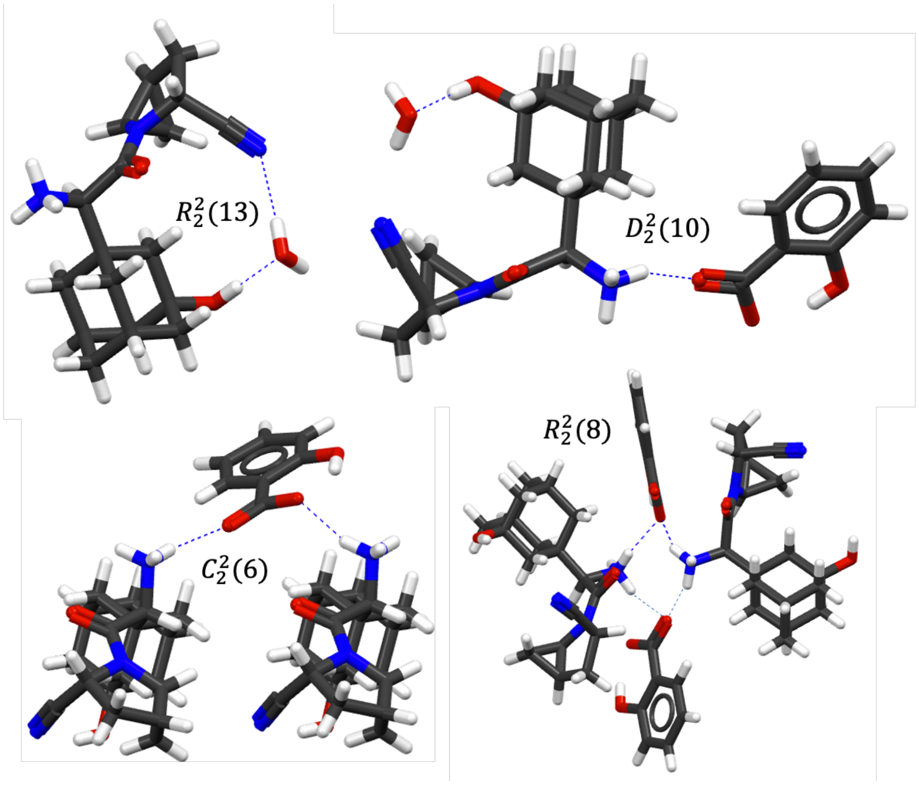

Figure S4. Selected graph sets for 2(**SAX**)•2(**SAL**)•(H_2O).

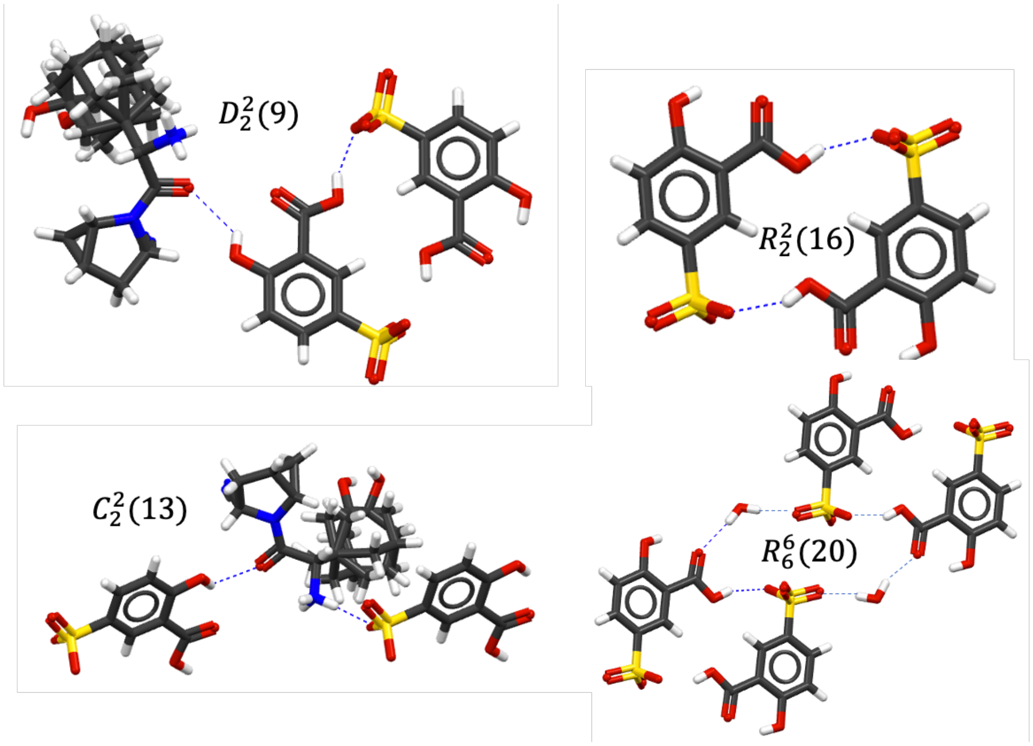

Figure S5. Selected graph sets for 2(**SAX**)•2(**SSA**)•2(H_2O).

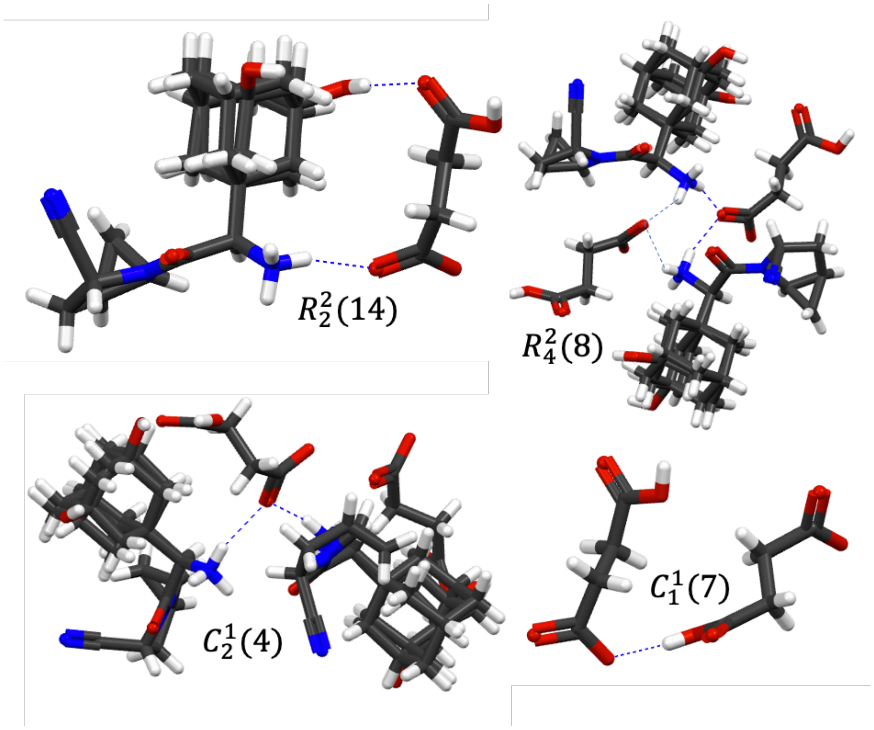

Figure S6. Selected graph sets for (**SAX**)•(**SUC**).

S3. Powder X-ray diffraction data

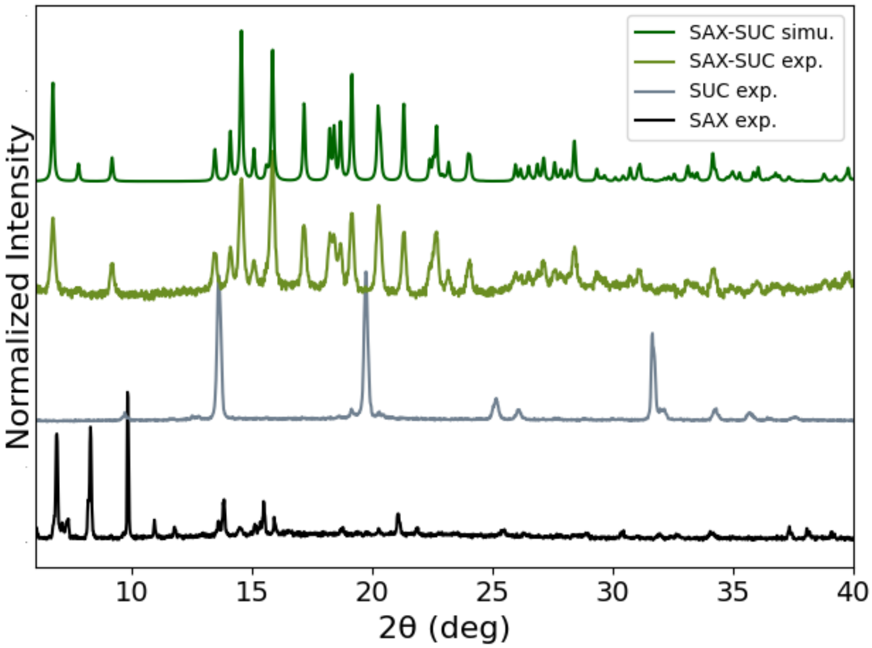

Figure S7. Powder X-ray diffractograms of (**SAX**)•(**SUC**) experimental and simulated from single crystal data, and starting materials.

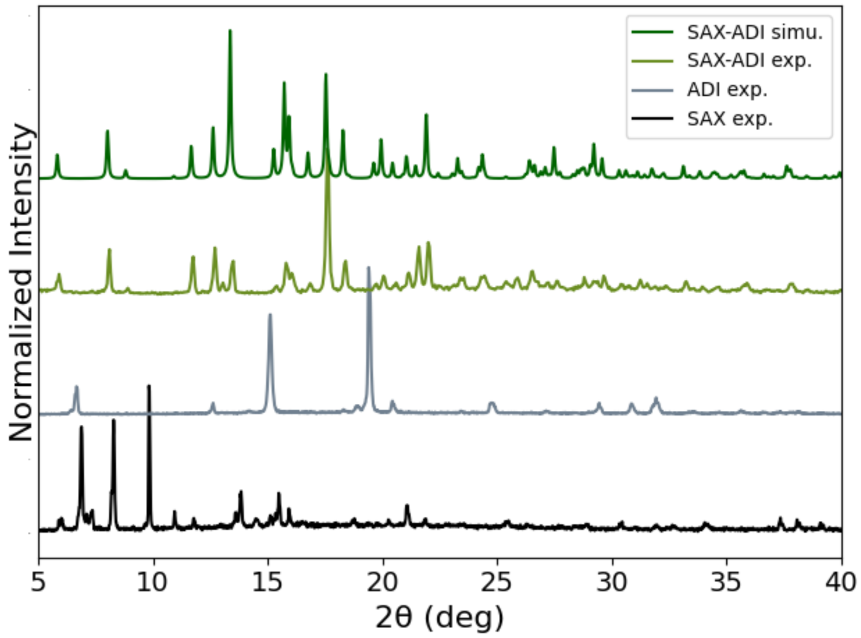

Figure S8. Powder X-ray diffractograms of 2(**SAX**)•(**ADI**)•6(H_2O) experimental and simulated from single crystal data, and starting materials.

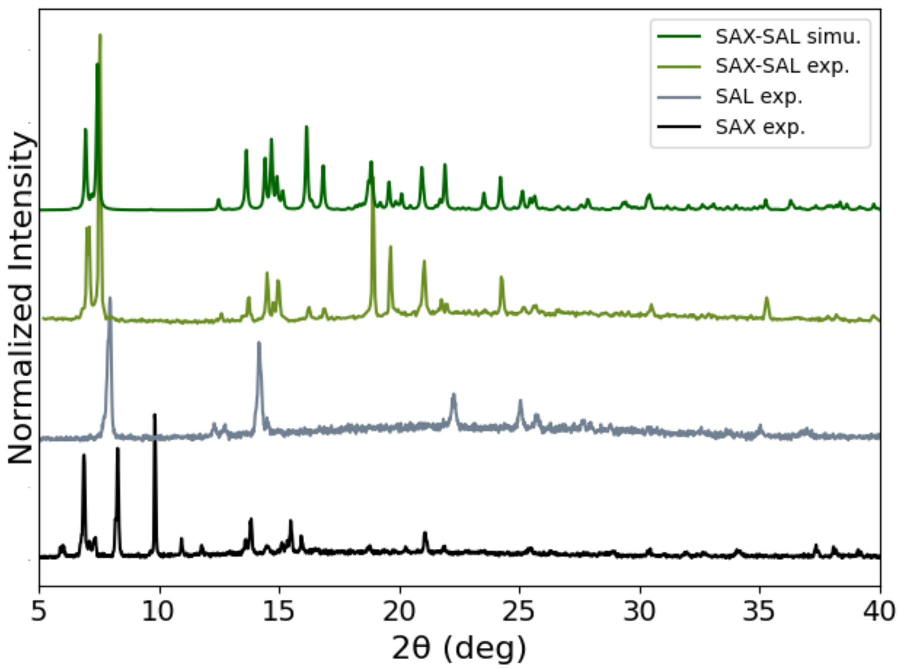

Figure S9. Powder X-ray diffractograms of 2(**SAX**)•2(**SAL**)•(H_2O) experimental and simulated from single crystal data, and starting materials.

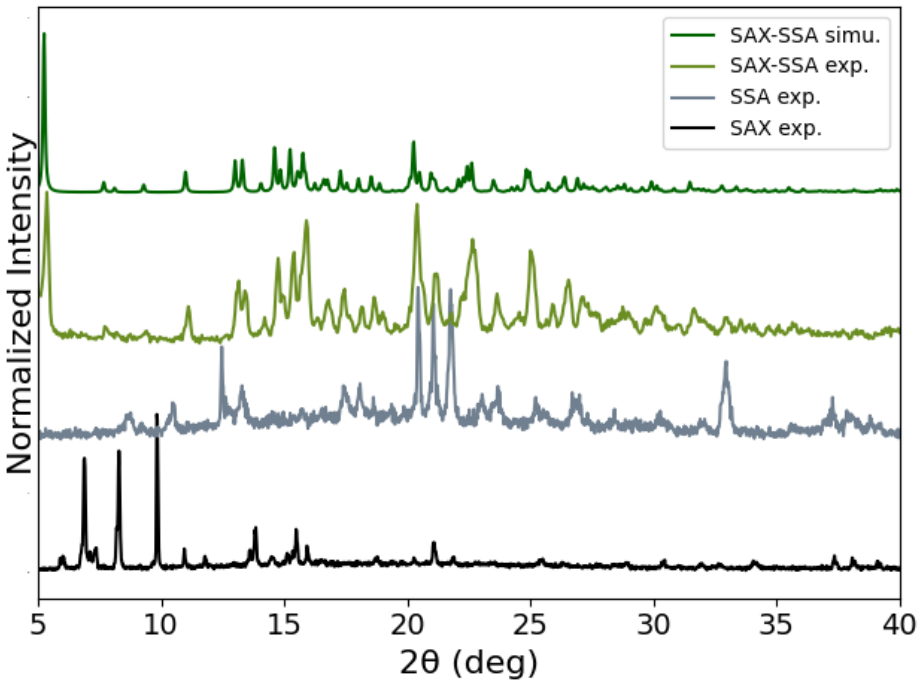

Figure S10. Powder X-ray diffractograms of 2(**SAX**)•2(**SSA**)•2(H_2O) experimental and simulated from single crystal data, and starting materials.

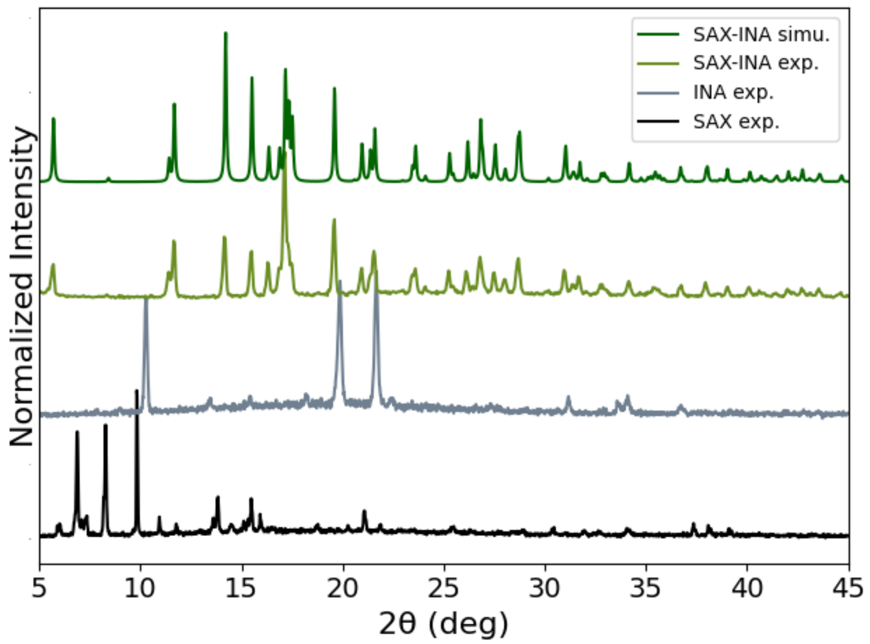

Figure S11. Powder X-ray diffractograms of (**SAX**)•(**INA**)•2(H_2O) experimental and simulated from single crystal data, and starting materials.

S4. FT-IR spectroscopy data

Table S7. Selected FT-IR absorptions for **SAX** multicomponent salts.

Salt	Observed ν (cm⁻¹)	Assignment
(**SAX**)·(**SUC**)	3394, 3273	ν(NH_3^+) stretch[1]
2(**SAX**)·(**ADI**)·6(H_2O)	3443, 3300	ν(NH_3^+) stretch
2(**SAX**)·2(**SAL**)·(H_2O)	3576, 3398, 3301	ν(NH_3^+) stretch
2(**SAX**)·2(**SSA**)·2(H_2O)	3516, 3409	ν(NH_3^+) stretch
(**SAX**)·(**INA**)·2(H_2O)	3394, 3273	ν(NH_3^+) stretch

[1]Broad bands in the 3300–3550 cm⁻¹ region correspond to stretching vibrations of the protonated amine (NH_3^+), consistent with charge-assisted hydrogen bonding in primary amine-.carboxylate salts. Minor shifts between salts likely arise from differences in hydrogen bonding environment and hydration.

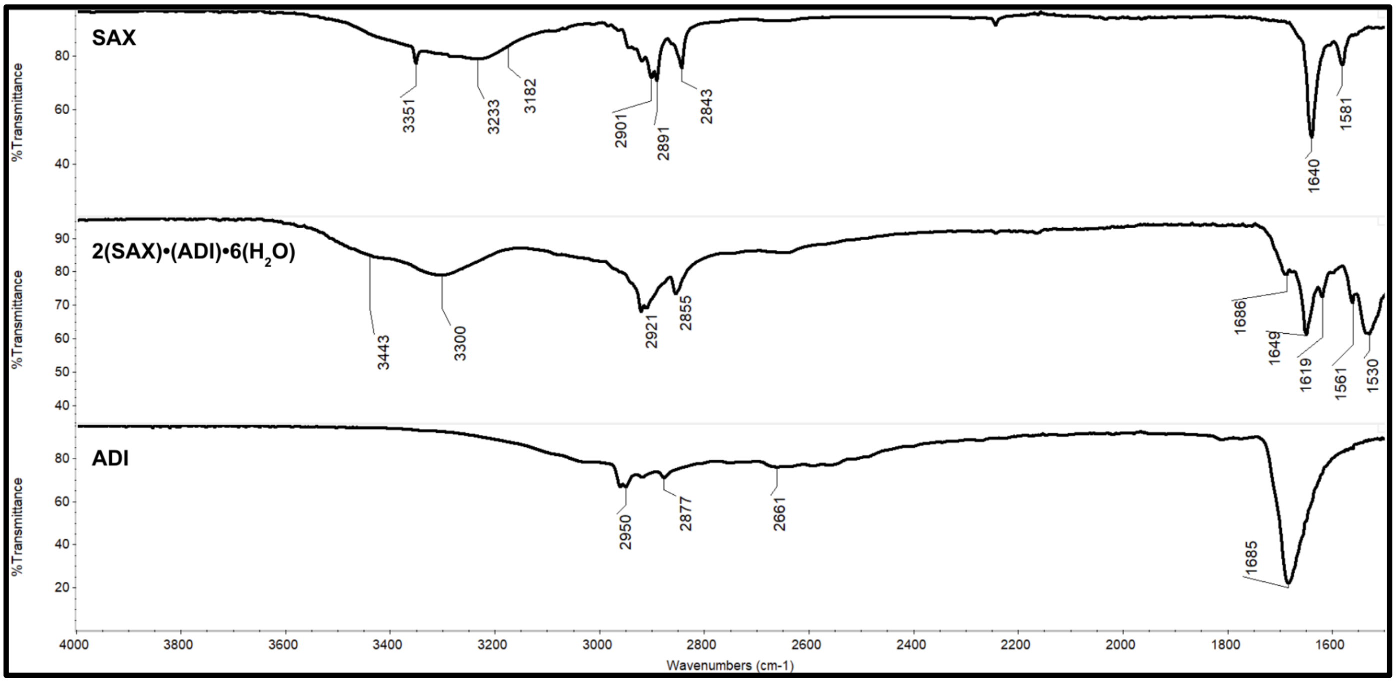

Figure S12. FT-IR spectrum of **SAX** and **ADI** starting materials and their resulting salt, 2**(SAX)•(ADI)•**6**(**H_2O**)**.

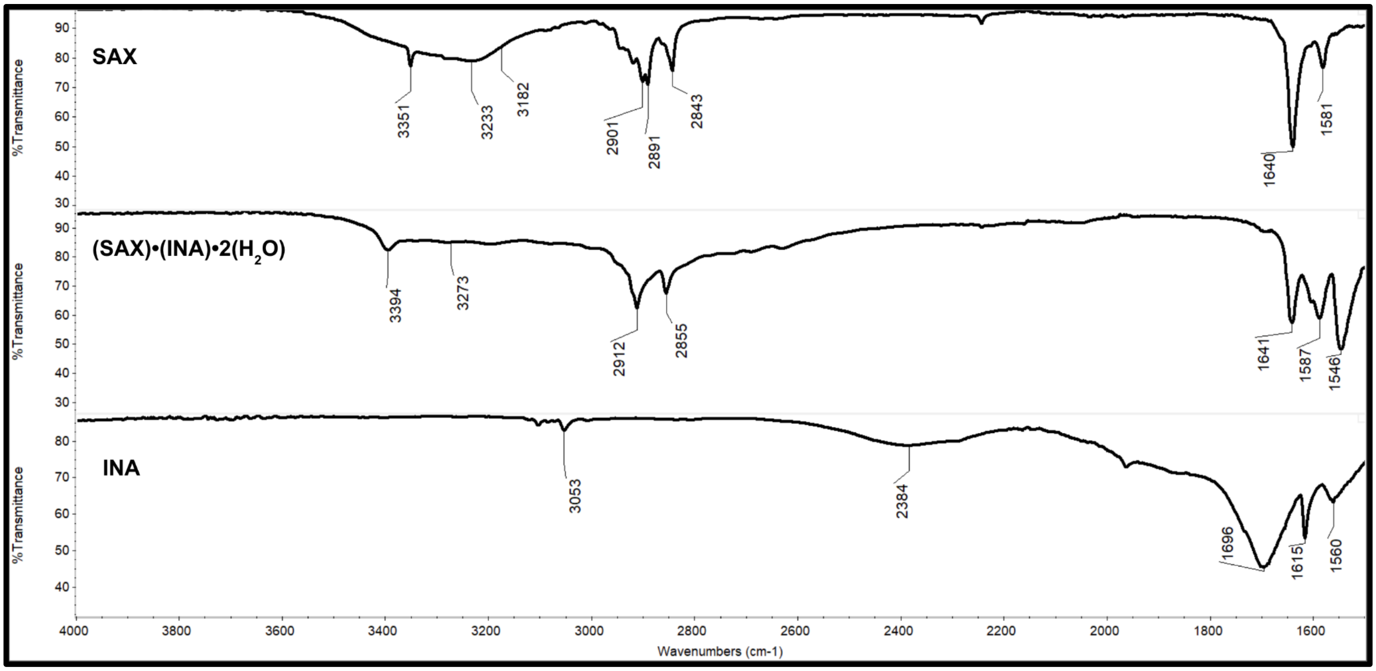

Figure S13. FT-IR spectrum of **SAX** and **INA** starting materials and their resulting salt, **(SAX)•(INA)•**2(H_2O**)**.

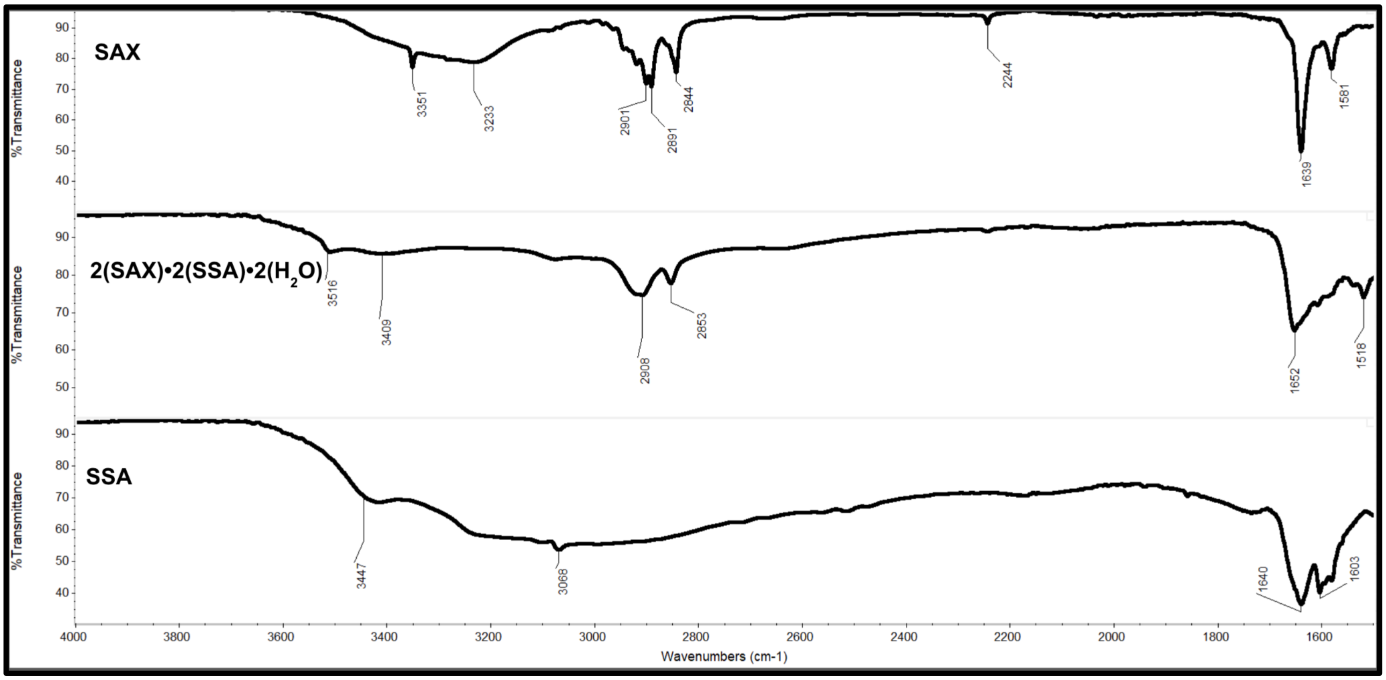

Figure S14. FT-IR spectrum of **SAX** and **SSA** starting materials and their resulting salt, 2**(SAX)•**2**(SSA)•**2(H_2O**)**.

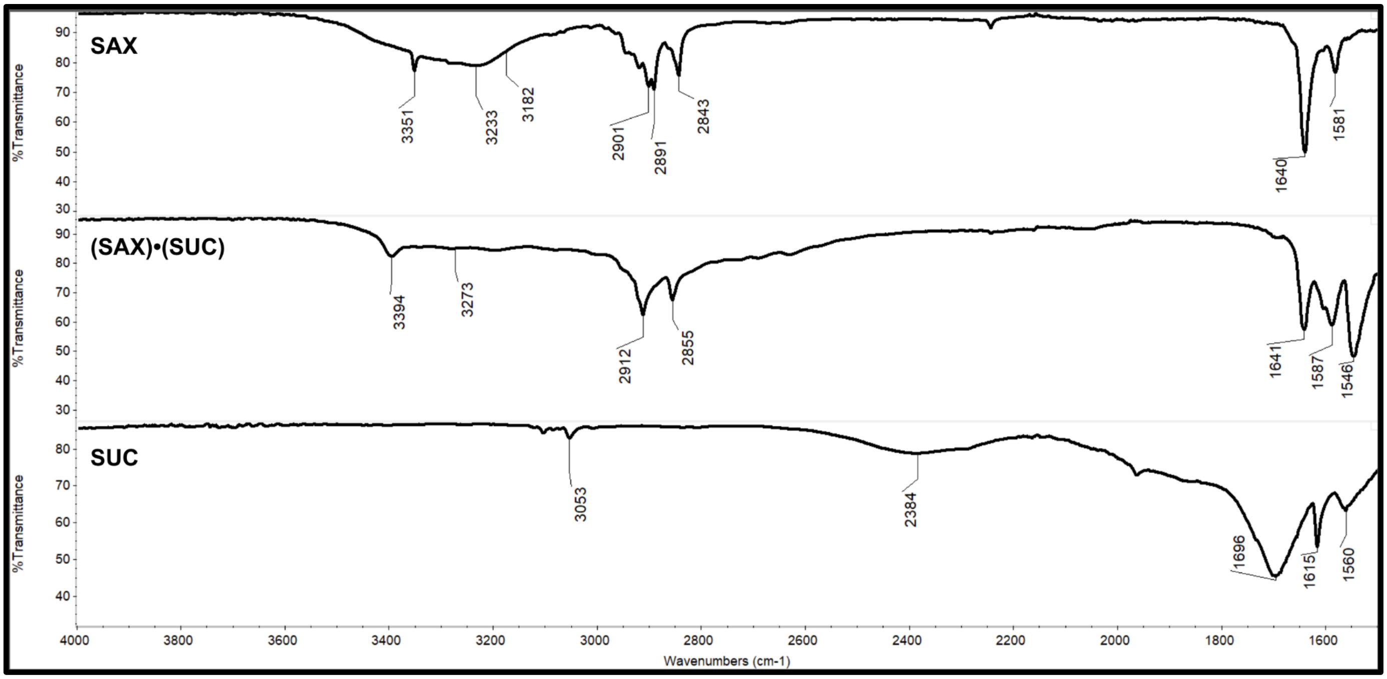

Figure S15. FT-IR spectrum of **SAX** and **SUC** starting materials and their resulting salt, **(SAX)•(SUC)**.

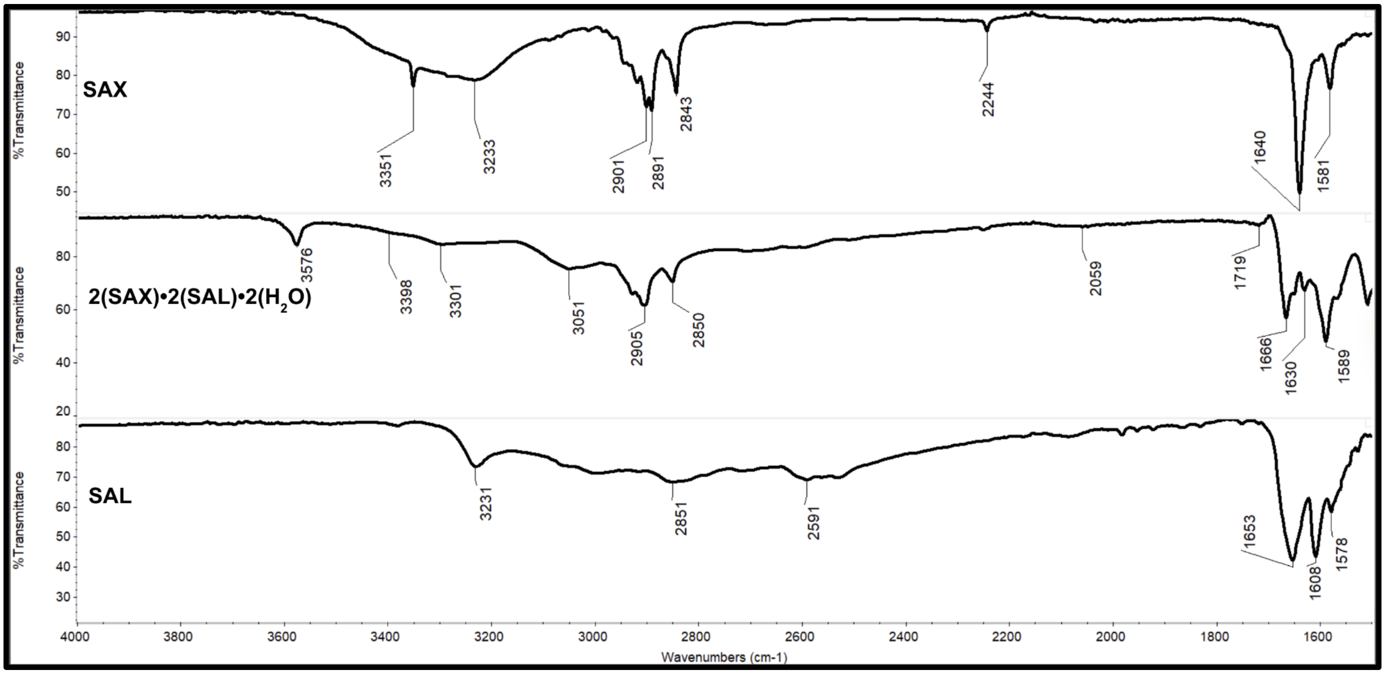

Figure S16. FT-IR spectrum of **SAX** and **SAL** starting materials and their resulting salt, 2**(SAX)•**2**(SAL)•**2(H_2O).

S5. NMR spectroscopy data

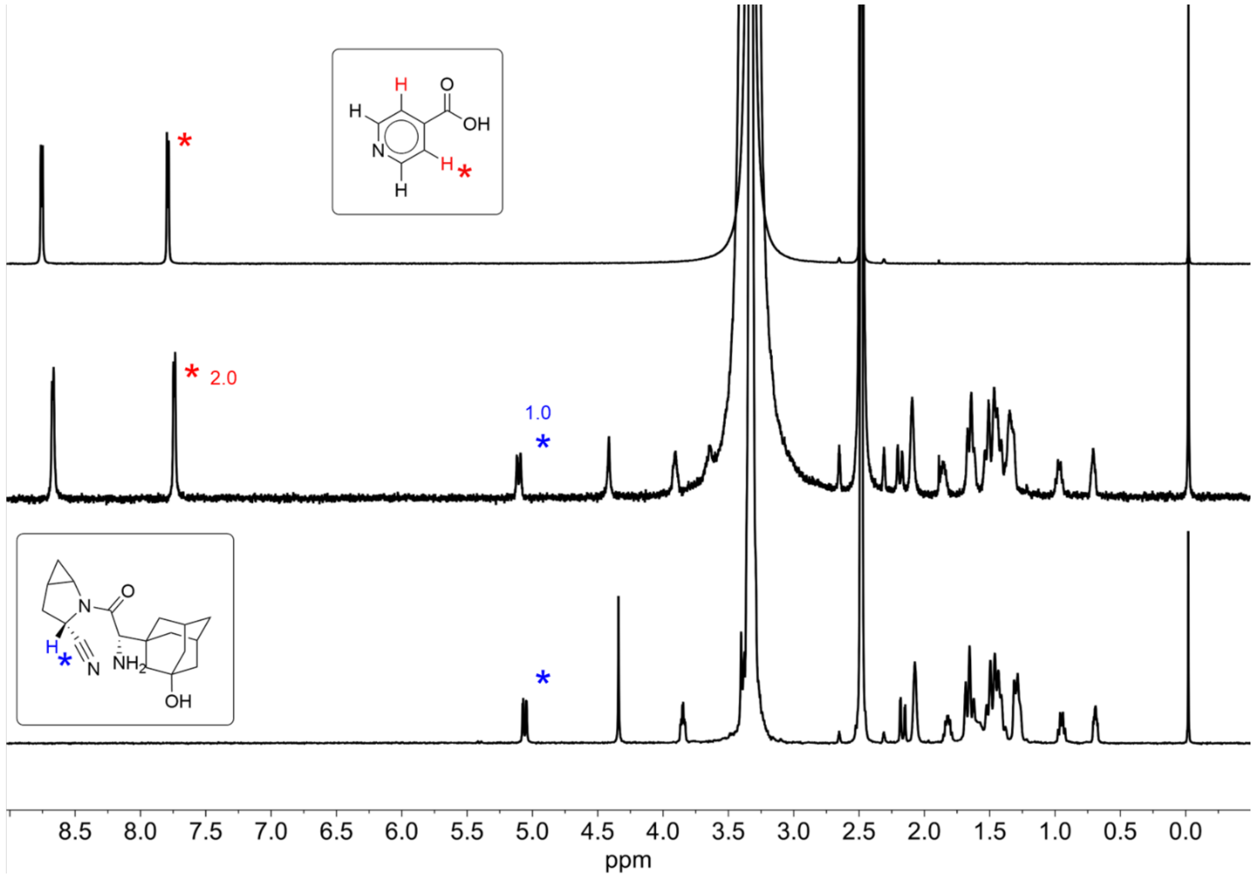

Figure S17. ^{1}H NMR spectra (400 MHz, DMSO-d$_6$) of **(SAX)•(INA)•2(H_2O)** and starting materials. Integration values for **SAX** and **INA** are given in blue and red, respectively.

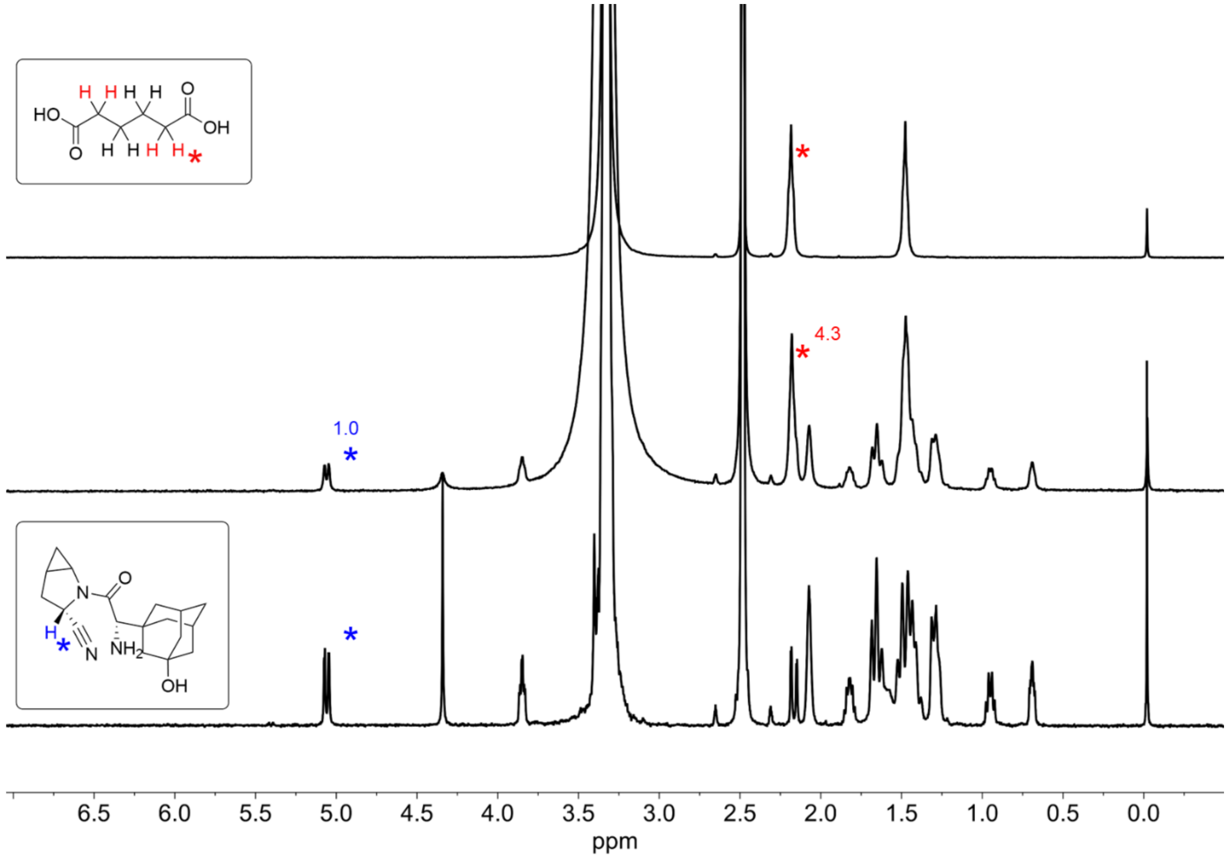

Figure S18. ^{1}H NMR spectra (400 MHz, DMSO-d_6) of 2**(SAX)•(ADI)•**6(H_2O) and starting materials. Integration values for **SAX** and **ADI** are given in blue and red, respectively. A larger value than expected of **ADI** is attributed to overlap with a neighboring peak.

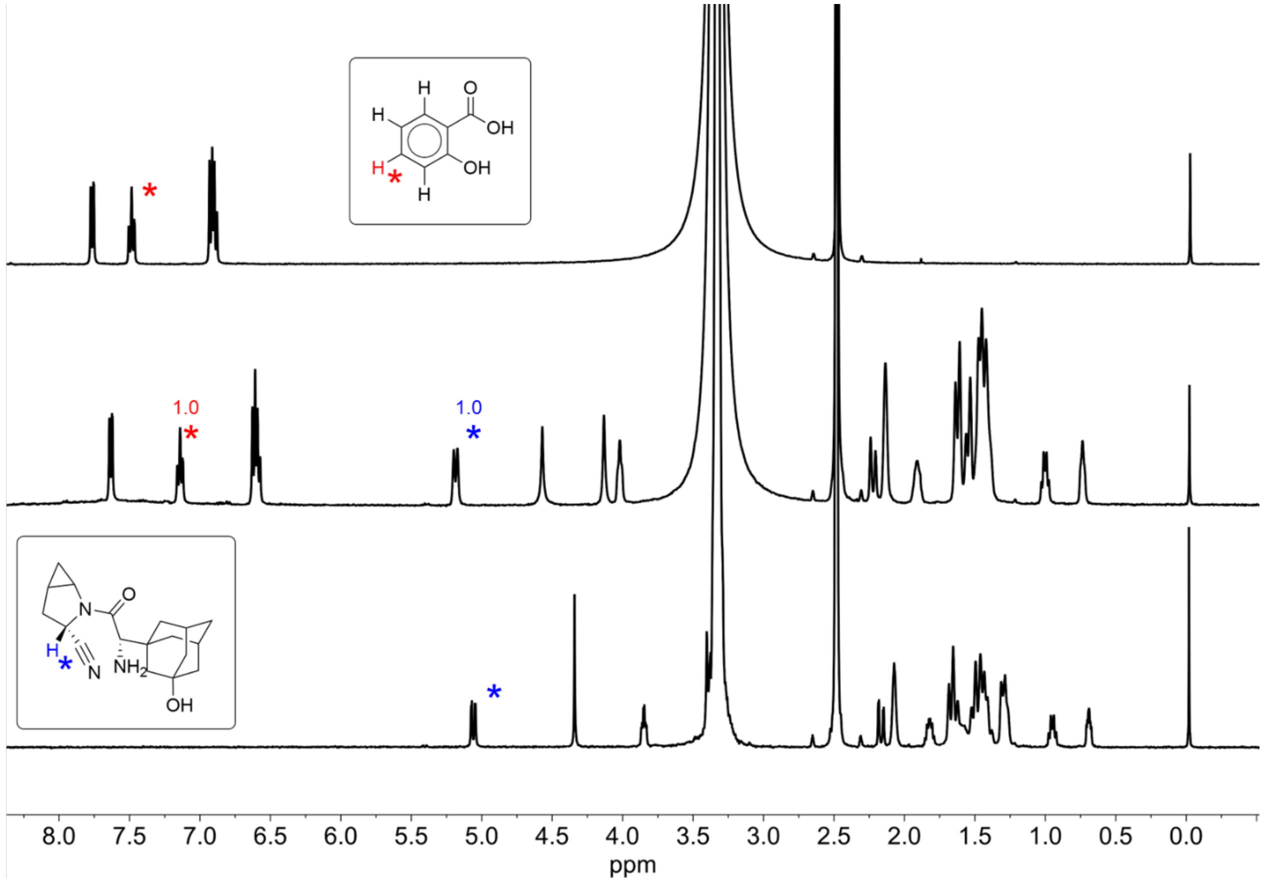

Figure S19. ^{1}H NMR spectra (400 MHz, DMSO-d_6) of 2**(SAX)**•2**(SAL)**•2(H_2O**)** and starting materials. Integration values for **SAX** and **SAL** are given in blue and red, respectively.

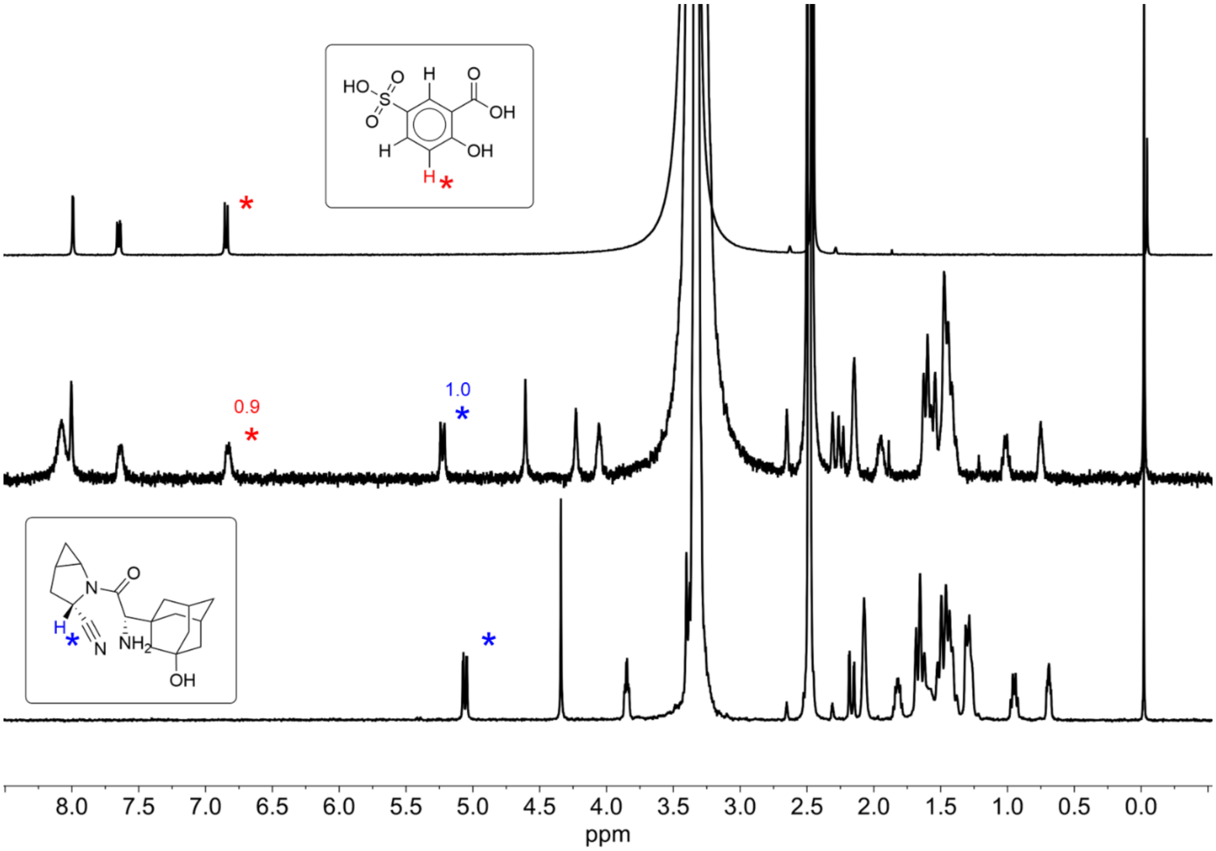

Figure S20. ^{1}H NMR spectra (400 MHz, DMSO-d$_6$) of 2**(SAX)**•2**(SSA)**•2(H_2O**)** and starting materials. Integration values for **SAX** and **SSA** are given in blue and red, respectively.

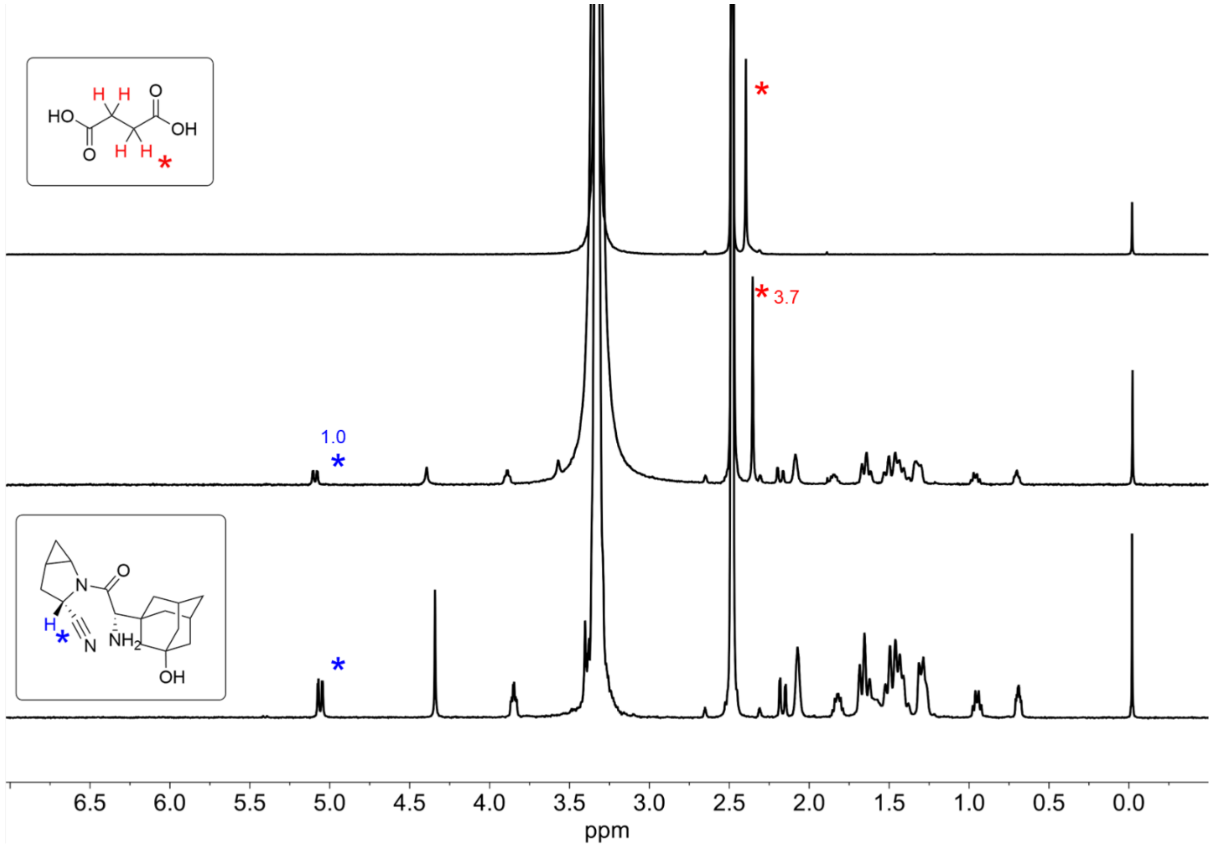

Figure S21. ^{1}H NMR spectra (400 MHz, DMSO-d$_6$) of **(SAX)•(SUC)** and starting materials. Integration values for **SAX** and **SUC** are given in blue and red, respectively.

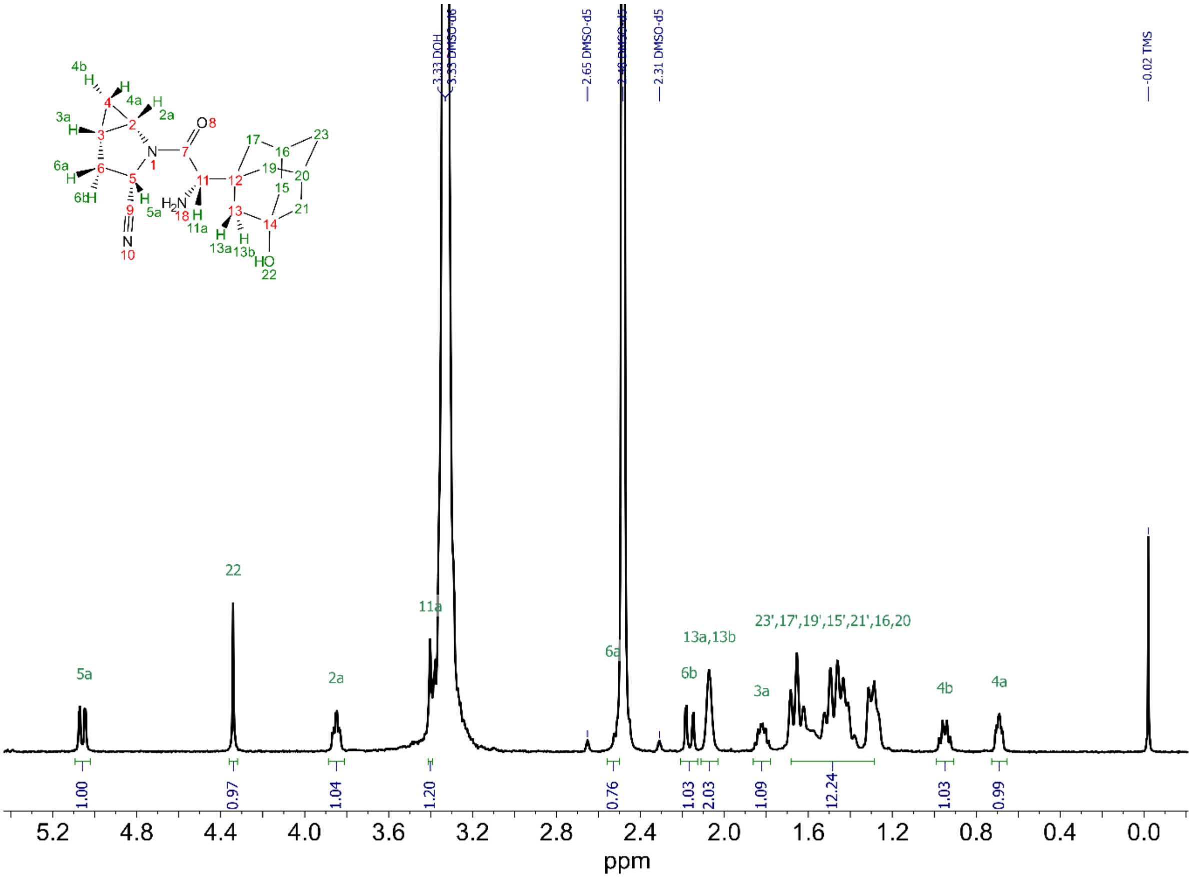

Figure S22. ^{1}H NMR spectrum (400 MHz, DMSO-d$_6$) of **SAX**.

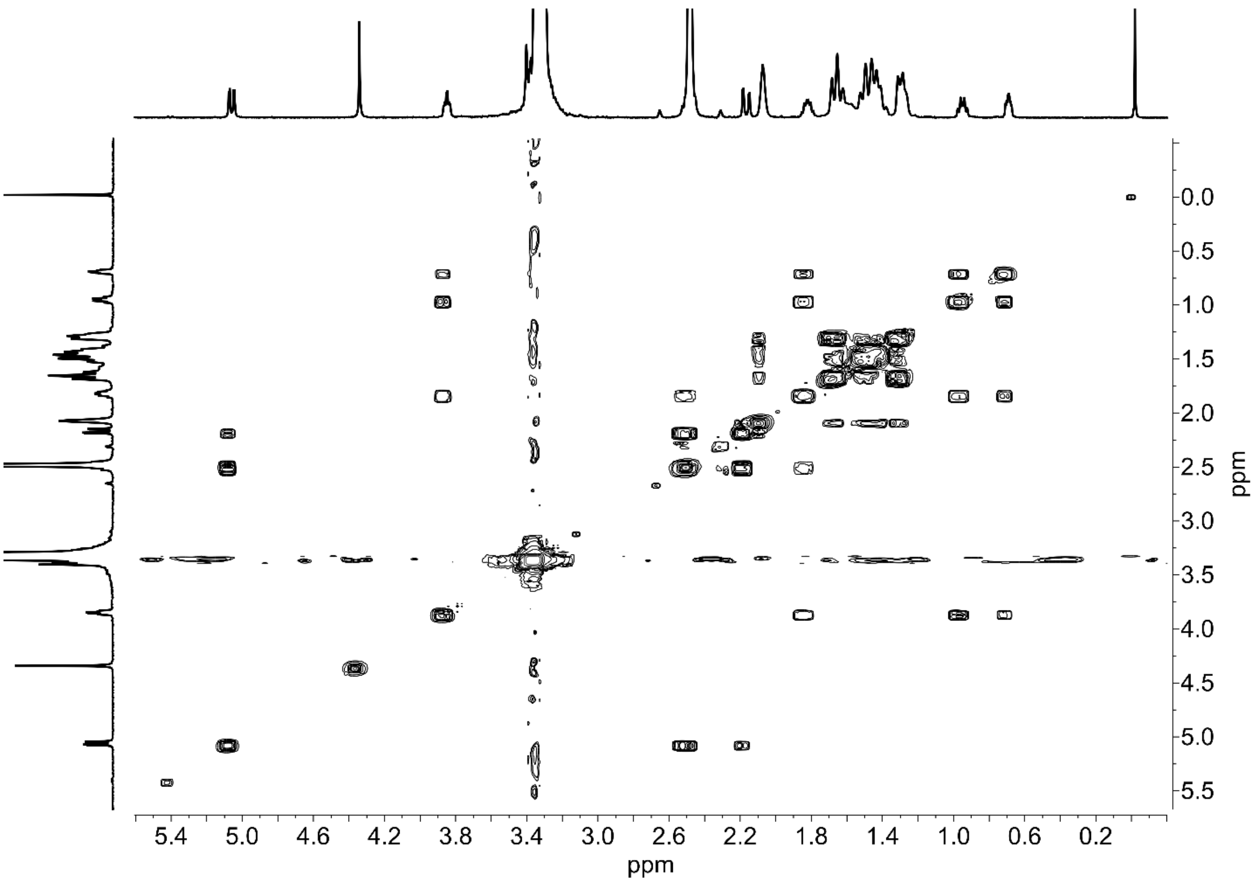

Figure S23. 2D ^{1}H-^{1}H COSY NMR spectrum (400 MHz, DMSO-d$_6$) of **SAX**.

References

(1) Gougoutas, J. Z.; Malley, M. F.; DiMarco, J. D.; Yin, X. S.; Wei, C.; Yu, J.; Vu, T. C.; Jones, G. S.; Savage, S. A. Crystal Forms of Saxagliptin and Processes for Preparing Same. US8802715B2, August 12, 2014.

(2) Marinkovic, M.; Ratkaj, M.; Frankovic, O.; Mundorfer, T. Polymorphs of Saxagliptin Hydrochloride and Processes for Preparing Them. US 8.410,288 B2, April 2, 2013.
